# Emerging Inkjet-Compatible Anti-Counterfeiting Inks Based on Microfluidic-Synthesized NIR PbS/CdS Quantum Dots

**DOI:** 10.1021/jacsau.5c00787

**Published:** 2025-09-18

**Authors:** Andi Magattang Gafur Muchlis, Chong-Ci Hu, Hoang-Duy Nguyen, Ramadhass Keerthika Devi, Yi-Ting Tsai, Yu Chun Lee, Chun Che Lin

**Affiliations:** † Institute of Organic and Polymeric Materials, 34877 National Taipei University of Technology, Taipei 10608, Taiwan; ‡ Research and Development Center for Smart Textile Technology, National Taipei University of Technology, Taipei 10608, Taiwan; § Institute of Advanced Technology, 61797 Viet Nam Academy of Science and Technology, Hochiminh City 700000, Viet Nam; ∥ Lextar Electronics Corporation, Miaoli 350402, Taiwan

**Keywords:** near-infrared, PbS/CdS quantum dots, microfluidic synthesis, inkjet printing, anti-counterfeiting

## Abstract

The lead sulfide (PbS) quantum dot (QD) material has excellent near-infrared (NIR) emissions. It is possible to synthesize core–shell-structured PbS/CdS QDs using the cation exchange method (Pb^2+^ → Cd^2+^), which enhances mass transfer and reaction uniformity and increases their stability. However, the large-scale synthesis of core–shell PbS/CdS QDs is needed to fulfill the industrial needs. Here, we used a microfluidic device to synthesize PbS and PbS/CdS QDs in enabling accurate control of flow rates, mixing, temperature, and residence time, which are critical for achieving uniform nucleation and controlled growth of QDs. This results in highly monodisperse nanocrystals with consistent optical properties. PbS QDs could be tuned by wavelengths from 1200 to 1600 nm and with full width at half-maximum (fwhm) varied from 213 to 272 nm. The core–shell PbS/CdS QD production was completed within 5 min, the wavelength was blue-shifted by 150 nm, and the fwhm was maintained at 223 nm with superior stability. The fabricated NIR QDs are then used to produce NIR emissive ink compatible with commercial-grade inkjet printers. An oily hydrophobic ink was produced by first dissolving the NIR QDs in an octadecene–octane mixture and then adding polyethylene to increase stability. After that, the ink was assessed by inkjet printing onto different paper substrates. Under the illumination of the blue light source, the printed ink was invisible under ambient and could be decrypted only when photographed by an NIR camera. This new NIR anti-counterfeiting identification mechanism represents a covert optical security approach, offering a novel, low-cost, and high-security solution for anti-counterfeiting applications.

## Graphical abstract

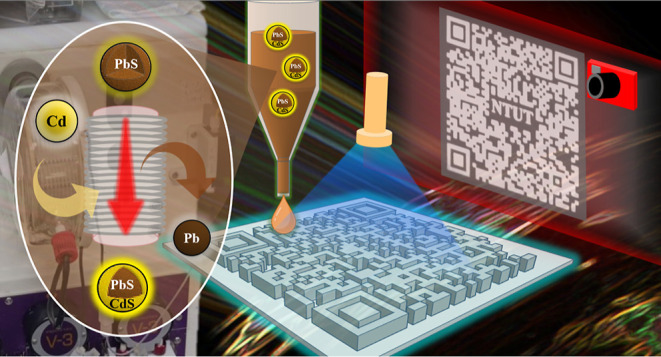

## Introduction

Near-infrared (NIR) quantum dots (QDs) have infinite potential in many applications. NIR QD applications include optical signal amplifier media for electrical communication systems.^1^ They are also often used in optoelectronic component applications,^2–11^ biomedical imaging, labels, and fluorescent sensing materials.^12–14^ As one of the NIR QDs with a wide range of adjustable energy gaps,^15^ a wide absorption range, and a Bohr radius up to 18 nm,^16,17^ lead sulfide (PbS) QDs are ideal candidates for NIR optoelectronic applications.^18^ For PbS QDs, considering the rapid nucleation and continuous growth steps, the most common synthesis method is the thermal injection method because of the advantages of low cost and simple preparation.^18^ Given these advantageous properties, developing efficient and controllable synthesis methods for PbS QDs has become a key research focus.

The synthesis process developed by Hines’s and Cademartiri’s teams is currently the most widely used method. Hines’s synthesis method used PbO and oleic acid (OA) as a lead precursor and a ligand, respectively, to form lead oleate under high temperature and inert conditions. After that, bis­(trimethylsilyl) sulfide (TMS) was used as a sulfur precursor. The sulfur–lead body is then injected into the lead oleate solution to synthesize PbS QDs.^19^ On the other hand, in Cademartiri’s synthesis method, PbCl_2_ was dissolved in oleylamine (OLA), and the sulfur precursor was rapidly dissolved in OLA. The sulfur–oleylamine precursor was swiftly injected into the lead solution and maintained at high temperatures to obtain PbS QDs.^20^

Those typical batch synthetic (hot injection) methods have the advantages of low process cost and simple operation. QDs are highly sensitive to certain conditions, such as reaction temperature, reaction time, and kinetics, and it is challenging to accurately regulate those aspects of synthesis. Therefore, it is easy to cause problems such as the uneven size of synthetic crystals and low experimental reproducibility.^21,22^ To overcome these limitations, microfluidic synthesis has emerged as a promising alternative.

The process of microfluidic synthesis is then expected to resolve these issues by applying a superior technology to traditional instruction synthesis. A microfluidic channel is a system in which the control fluid is spatially confined to a small scale (usually 1 to 0.1 mm).^23,24^ Therefore, we make a novel synthesis system for QDs utilizing microfluidic synthesis, which has advantages such as an excellent mixing effect, good handling of high heat and mass transfer, enabling production of high-specific-surface-area QDs , more precise temperature control, continuous production, and reducing reagent usage.^25–28^ Therefore, using this method enables the synthesis of QDs with a relatively higher scale in a short time.

Several research groups have demonstrated the potential of this approach. In 2013, Bakr’s team synthesized PbS QDs through a two-stage reactor flow channel system.^29^ In 2014, the deMello team synthesized PbS QDs by feeding two lead and sulfur precursors with a background solution into a cross-connector and a Teflon tube, which was immersed in an oil bath using three separate syringes.^30^ In 2015, the deMello team investigated the early kinetics of PbS QD production using a unique droplet-based microfluidic technology.^31^ However, we found that PbS QDs are unstable, and air readily oxidizes them. Therefore, the core–shell structure was developed to improve the PbS QD stability. The formation of shells for the PbS core using CdS by cation exchange is the most commonly used method because the surface Pb^2+^ cations can easily exchange with the Cd^2+^ cations in the solution. The shell growth then occurs at the expense of the core crystal size. The shell growth is accompanied by the PbS core size reduction, resulting in an emission spectral blue shift.^32–40^

Furthermore, an advancement has been proposed to produce PbS/CdS core/shell QDs with tunability to NIR wavelength reaching 1600 nm using the batch method assisted with microwave irradiation.^36^ Moreover, adapting continuous flow synthesis that employs the concept of microfluidic synthesis, Machut et al. (2024) successfully demonstrated the scalable synthesis of PbS/CdS QDs with tunable NIR emission at approximately 1100 nm.^41^ Their approach served as an inspiration for this study’s goal, which is to further explore and optimize the reaction parameters with a focus on reducing reaction time while maintaining desired optical properties. In our synthesis, we purposefully used TMS as a sulfur precursor because of its exceptional reactivity and suitability for rapid, regulated nanocrystal growth. Although their method typically takes 10–20 min to tune the emission wavelength, our results show that, under optimal conditions, comparable NIR emission can be obtained starting from 5 min.

Beyond stability improvements and rapid, big scale preparation potential, the unique NIR tunability of PbS/CdS QDs opens up exciting opportunities in emerging applications such as anti-counterfeiting. The increasing problem of counterfeiting security and confidential documents, like money, passports, national identification cards, certifications, and valuables data, makes data protection through anti-counterfeiting technology essential.^42^ One of the best anti-counterfeiting identification platforms is photoluminescence, which is invisible to the human eye but can reveal confidential data when exposed to ultraviolet or visible light.^43^ However, in this field, visible light emission is commonly used, and there is a lack of research that used NIR QDs for anti-counterfeiting application. NIR light is invisible to human naked eyes but can be captured by an NIR camera. This advantage will be used to give better security of information; thus, a new security system can be invented.

To ease the application of luminescent materials like NIR QDs in the anti-counterfeiting application, the printability and customizable luminescent properties of luminescent materials have attracted the intense attention of researchers.^44^ Inkjet printing, because of its high resolution, large area, and low-cost fabrication,^45,46^ is an excellent way to realize luminescent anti-counterfeiting. Various visible light luminescence QDs have been developed in the anti-counterfeiting identification field with different new luminescence mechanisms and signals, even to perform reversible luminescence properties.^47–52^ However, fabricating a suitable formulation of QD inks that are suitable for inkjet printing is challenging since we need to consider the viscosity, surface tension, density, and boiling point to the printed papers. Therefore, this work also proposes an optimalization of NIR QD ink composition for inkjet printing application suitability.

Overall, this research aims to use a microfluidic system to synthesize PbS/CdS QDs that could shorten the reaction time and control the temperature to synthesize the core/shell QDs’ structure with high stability. After NIR QDs were prepared, the ratio of 1-octadecene (ODE) to *n*-octane additive solvents could be adjusted to obtain the desired ink characteristics for printing by commercial printers. Ink stability was increased by the addition of a polyethylene (PE) polymer. The formulated NIR QD ink was printed on different paper substrates to assess its printability.

Also, we introduce the improvised encryption method to double-protect the security of the encrypted data. After fabrication, the NIR ink was used in inkjet printing to produce a pattern that is invisible to the naked eye. A blue light source was shining on the encrypted printed paper, but no visible light emission was seen. The printed paper must be photographed by an infrared camera to decrypt the printed NIR QD graphics or text, achieving high-security anti-counterfeiting application. Therefore, a novel NIR anti-counterfeiting identification application was proposed in this work and is briefly described by Scheme 1.

**Scheme 1:**
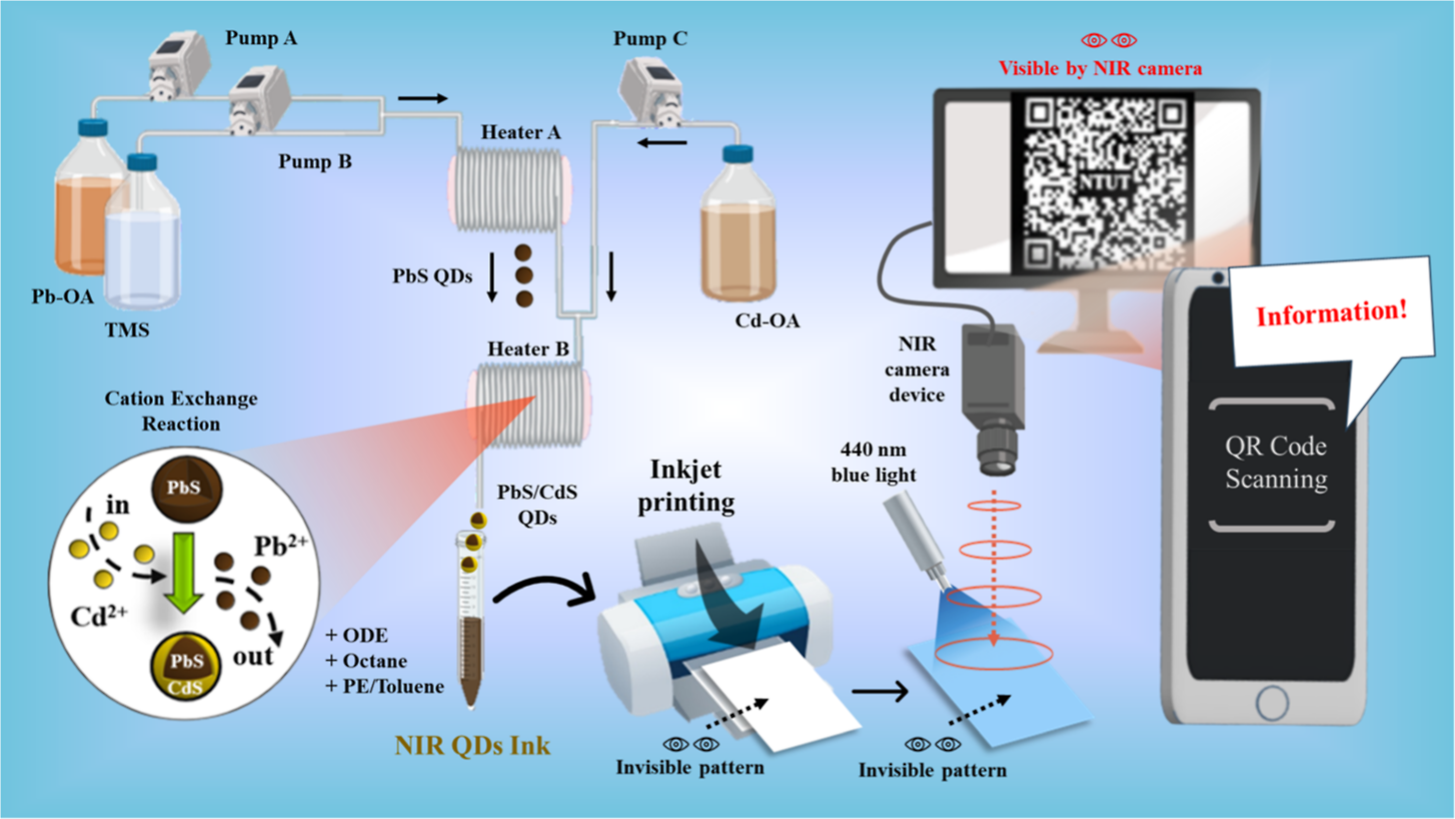
A Schematic Diagram of the Synthesis of PbS and PbS/CdS QDs Using a Microfluidic System Prepared into an NIR Ink Utilizing Additive Solvents and a Polymer, Followed by Inkjet Printing on Paper and NIR Anti-Counterfeiting Identification

## Experimental Section

### Materials and Reagents

We purchased and further used lead oxide (PbO, 99.9%; Acros Organics), bis­(trimethylsilyl) sulfide (TMS, 95% purity; TCI), cadmium oxide (CdO, 99%; Acros Organics), 1-octadecene (ODE, 90%; Acros Organics), oleic acid (OA, 99%; Sigma-Aldrich), toluene (99.5%; Acros Organics), octane (98%; Alfa Aesar), ethanol (95%; ECHO), tetrachloroethylene (TCE, 99%; Alfa Aesar), and polyethylene (PE (Mw ∼ 35,000; Sigma-Aldrich). All chemicals were used as received without purification except for ODE. We used ODE as a TMS stock solvent, which was used after heating under vacuum in advance.

### Characterizations

The photoluminescence (PL) spectra of PbS and PbS/CdS QDs were measured with an IHR320 spectrophotometer (HORIBA) at an excitation wavelength of 440 nm. The UV–vis absorption spectra of PbS and PbS/CdS QDs were measured with an IHR320 spectrophotometer (HORIBA). A tungsten filament bulb was used as a light source, and a current controller (GW Instek GPR-30H100) was used to control the power of the tungsten filament bulb to control the voltage to 300 W. The structure and crystal phase of the PbS and PbS/CdS QDs were characterized by X-ray powder diffraction (XRD, Bruker D8, Cu Kα radiation, λ = 1.54058 Å). The particle size and morphology of PbS and PbS/CdS QDs were characterized by transmission electron microscopy (TEM, JEOL-2100F). Fourier transform infrared (FTIR) spectra were obtained on an FTIR spectrometer (Spectrum One, PerkinElmer). The viscosity of the ink was measured by an Ostwald viscometer (026300-1, SIBATA). The surface tension of the ink was measured by using a digital surface tension meter (CBVP-Z, Kyowa Interface Science). The contact angle of the ink was measured by a contact angle tester (Phoenix 300, APPLIED MICROTECH Inc.).

### Preparation of Pb-OA and S Precursors

For the Pb-oleate (Pb-OA) precursor, 0.361 g of PbO, 20 mL of ODE, and 2 mL of OA were added to a 50 mL three-necked flask. These chemicals were kept under vacuum at 110 °C for 1 h until PbO had reacted with OA completely. Furthermore, it was heated from a yellow powder to a transparent Pb-oleate solution. After that, we stirred the solution under a nitrogen gas flow until the solution was cooled to room temperature. For the TMS precursor, we mixed 168 μL of TMS with 30 mL of dried ODE in a glovebox.

### Synthesis of PbS QDs via the Microfluidic System

We applied the microfluidic system from Vaportec Co., Ltd. with the product model of the Vaportec easy-MedChem flow chemistry system (Figure S1a). Peristaltic pumps’ input pipes were wetted into the bottles containing both precursors before the synthesis. According to the program, the precursors would be pulled simultaneously into the microfluidic system with a Pb/S = 1:1 ratio. All of the flow rates were fixed at 2.5 mL/min, the reaction times were set at 2 min, and the temperature of the reaction was adjusted between 90 and 150 °C. The product was gathered once the reaction was finished. The collected product was added to toluene and ethanol for purification purposes and was centrifuged at 7000 rpm for 5 min, and then the precipitated PbS QDs were dissolved in toluene for further use.

### Synthesis of PbS QDs via the Hot Injection Method for Comparison (Batch Synthesis)

The same method was used to prepare the phosphate oleate (Pb-OA) precursor. After backfilling the system with nitrogen, Pb-OA solution was heated to 90 to 150 °C. 168 μL aliquot of TMS solution with 4 mL of dried ODE solution was swiftly injected into the heated Pb-OA. The reaction time was set to 2 min, after which it was cooled to room temperature with an ice bath. The same purification method was used to obtain a precipitate of PbS QDs, which was then dissolved in toluene or TCE.

### Preparation of PbS and Cd-OA Precursors for the Synthesis of PbS/CdS QDs

For the Cd-oleate (Cd-OA) precursor, 0.8859 g of CdO, 30 mL of ODE, and 6 mL of OA were added in a 50 mL three-necked flask; these chemicals were kept under N_2_ at 255 °C for 20 min until CdO had totally reacted with OA (from a red powder to a transparent Cd-oleate solution). After that, we stirred the solution under vacuum until it cooled to 110 °C for around 20 min. Furthermore, the mixture was cooled again to 100 °C under a N_2_ flow. The Cd-OA precursor solution was stored in a 50 mL three-necked flask for the later synthesis in the microfluidic system. For the PbS precursor, the PbS QD suspension in toluene (3 mL, absorbance = 3 at the first exciton peak) was diluted in 30 mL of toluene and sonicated for 30 min. The PbS precursor solution was stored in a 50 mL three-necked flask for the later synthesis in the microfluidic system.

### Synthesis of PbS/CdS QDs via the Microfluidic System

Before the synthesis, the inlet pipes of peristaltic pumps were wetted with both precursors’ bottles first. The precursors would be drawn into the microfluidic system at the same time with a PbS/CdOA = 1:1 ratio according to the program. The flow rates were all set at 1–0.2 mL/min, reaction times were 5–20 min, and reaction temperature was 100 °C. After the reaction was complete, the product was collected. The collected product was added to toluene and ethanol for purification purposes and was centrifuged at 7000 rpm for 5 min. Then, the precipitated PbS/CdS QDs were dissolved in a solvent (toluene or TCE) for further use.

### Preparation of PbS/CdS Ink for Inkjet Printing

The as-synthesized PbS/CdS QDs were diluted (20 mg/mL) with mixed binary solutions of ODE and octane at different volume ratios. On the other hand, a PE/toluene suspension was prepared by adding PE to 5 mL of toluene (ranged 0.025–0.125 g). The reaction temperature was set to 90 °C for 30 min until PE was completely dissolved. After adding PE/toluene solvent to the octane/ODE binary solvent, we could obtain the octane/ODE/PE/toluene ternary solvent. The inkjet printing machine we used was from EPSON with model L121 (Figure S1b). The self-modified printer cartridge was used instead of the original one. We used a standard black ink cartridge that was thoroughly cleaned, and the PbS/CdS QD ink was refilled into this cartridge for inkjet printing. The NIR QD anti-counterfeit patterns were printed out on different types of papers.

### NIR Anti-Counterfeiting Identification Application

We utilized an infrared camera from ISUZU OPTICS CORP with the product model Ninox 640 II VIS-SWIR (Figure S1c). We installed a visible light filter in front of the lens and fixed the infrared camera at 30 cm on the table. The anti-counterfeiting pattern of NIR QDs was printed on paper and placed on the desktop. The printed pattern was irradiated with a blue flashlight (λ = 440 nm). The camera captured the images, which we then transferred to a computer so that we could store them. The use of long-wavelength luminescence beyond the visible range, combined with the compatibility of the ink formulation for commercial inkjet printing, offers a novel strategy for high-security authentication. The QDs’ core–shell architecture, formed via controlled cation exchange, enhances photostability and minimizes surface oxidation—two critical factors for prolonged ink performance. The system demonstrates synergy, photonic response tuning, and scalable device fabrication, enabling invisible, NIR-readable information encoding for next-generation security technologies.

## Results and Discussion

### Structural Characteristics of PbS QDs

Figure 1a shows the XRD pattern of PbS synthesized at 90–150 °C using a microfluidic system. The microfluidic system produces PbS QDs with a consistent crystal phase across 90–150 °C temperature range, showing XRD pattern that match the reference and those obtained from the batch synthesis method (Figure S2). This result indicates that this microfluidic system can reliably reproduce PbS QDs, as typically achieved by batch methods, across different temperatures.^53^ In addition, we determined the average value of the full width at half-maximum (fwhm) from the XRD patterns of the synthesized PbS QDs of the microfluidic system, and the particle size was calculated by the Scherrer equation, which was defined as eq 1:^54^

1 D=Kλ/βcos⁡θ

where *D* is the crystallite size in the direction perpendicular to the lattice planes, *K* is the Scherrer constant (0.94), λ is the X-ray wavelength (1.54058 Å), β is the width of the X-ray diffraction peak in radians, and θ is the Bragg angle. The particle sizes of PbS QDs (synthesized using microfluidic and batch synthesis) based on their XRD fwhm are displayed in Tables S1 and S2, and the particle size comparison is plotted in Figure S3. We calculated the average particle sizes derived from three main peaks, 26°, 30.1°, and 43.1°, in the XRD pattern.

**Figure 1:**
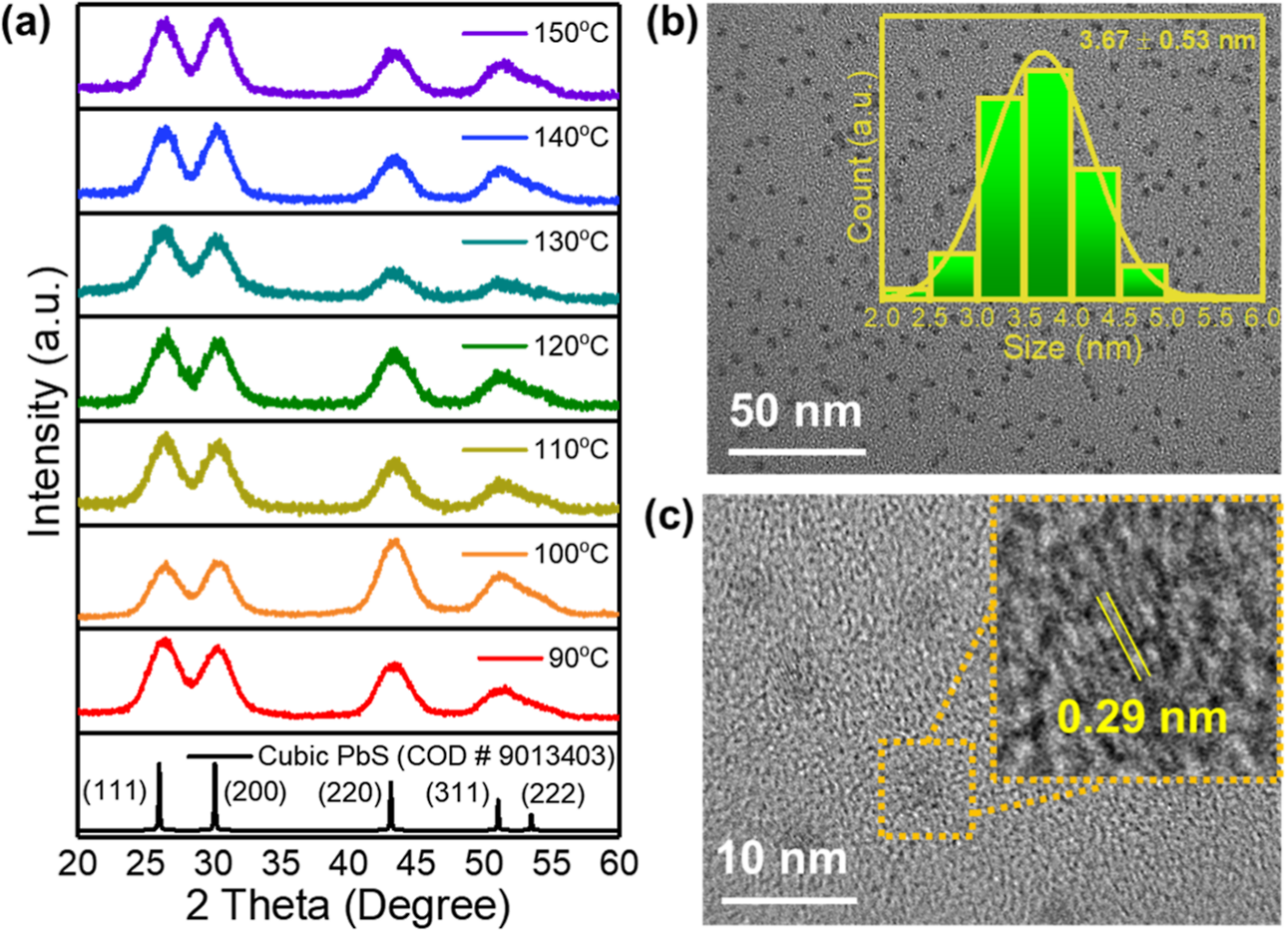
(a) XRD patterns of the microfluidic-synthesized PbS QDs. (b) TEM image of the microfluidic-synthesized PbS QDs (inset: TEM image-derived particle size distribution of the microfluidic-synthesized PbS QDs). (c) HRTEM and lattice fringe images of the microfluidic-synthesized PbS QDs.

Figure S3 illustrates that the particle size of PbS synthesized using the hot injection method and microfluidic method (based on XRD data) ranged approximately from 4 to 6 nm with a slight difference. This result indicates that nanosized PbS QDs were successfully synthesized by these two methods and signified that the microfluidic method we proposed was reliable enough. Figure 1b exhibits the morphology of the PbS QDs synthesized by the microfluidic system that was observed via TEM imaging. The result reveals the success of synthesis with fulfilling QDs dots’ morphology with the expected nanoscale dimension. Figure 1b (inset) shows that the diameters of the prepared PbS QDs were 3.67 ± 0.53 nm, confirming the narrow size distribution of the synthesized PbS QDs using the microfluidic system synthesis. From the HRTEM image, we can analyze the spacing between the lattice fringes calculated as 0.29 nm, which refers to the cubic structure of the (2 0 0) PbS lattice plane (Figure 1c).^55^ Overall, the characterizations show that the method we used to make PbS QDs is as reliable as the batch synthesis method and even has advantages in easy controlling parameters and upscalability.

### Optical Properties of PbS QDs

Figure 2a,b shows the PL spectra of PbS QDs synthesized by a microfluidic system with different temperature conditions. They show the fluorescence emission and absorption spectrum of PbS QDs that are shifted to longer wavelengths by the increase of the reaction temperature. For a better comparison, Figure 2c implies that the emission wavelength can be adjusted from around 1200 to 1500 nm by increasing the reaction temperature. Also, as the reaction temperature elevates, the fwhm of the fluorescence emission spectrum is broadened, which is consistent with the literature reports by deMello et al., who believed that higher temperatures may lead to the formation of bigger particles and make the size distribution nonuniform.^30^ The fluorescence emission and absorption spectra of PbS QDs synthesized by the batch hot injection method are shown in Figure S4.

**Figure 2:**
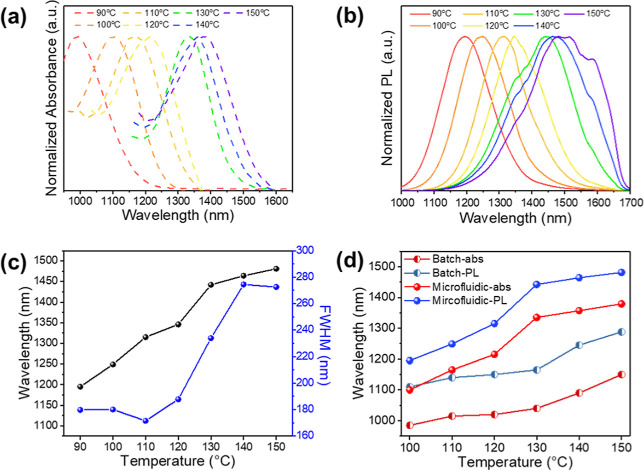
(a) Absorption and (b) emission spectra of PbS QDs synthesized by the microfluidic system at different reaction temperatures. (c) Comparison graphic of emission wavelength and fwhm of PbS QDs synthesized by the microfluidic system at different temperatures. (d) Comparative diagram of PbS QDs’ spectral wavelengths produced using batch and microfluidic systems at various reaction temperatures.

The PL emission and absorption wavelength change of PbS QDs synthesized by the microfluidic system and batch system are compared, as delivered in Figure 2d. The spectral wavelength of the synthesized PbS QDs by the microfluidic system is more inclined to higher wavelengths over reaction temperature compared to that by the batch system.^56^ In the batch system, the temperature needs to be adjusted from 120 to 190 °C to achieve an emission wavelength from 1100 to 1600 nm (Figure S5). In contrast, the microfluidic system can achieve that at a range of 80–150 °C with relatively neater intervals (Figure 2a,b). We believe that this better efficiency is caused by proper mixing, accurate temperature, high heat and mass transfer, and a high surface-to-volume ratio offered by the microfluidic system.^27^ Heat and mass transfer and fast diffusion rates should saturate faster than by prescribed synthesis, and precise temperature control may provide higher reaction rates for fast nucleation and growth.^28,57,58^ Therefore, we conclude that this microfluidic method can improve PbS QDs’ optical properties’ adjustable efficiency compared to the batch hot injection method. Although the fwhm and average particle sizes from the two synthesis techniques are comparable, the QDs produced using the microfluidic system typically show a marginally wider fwhm. The continuous nature of the microfluidic synthesis and minute fluctuations in growth rates could be the cause of this wider spectral width. For multiwavelength anti-counterfeiting applications, this may result in improved overlap, more severe emission intensity, and increased NIR absorption.

### Core/Shell PbS/CdS QD Characteristics

Due to PbS susceptibility to environmental oxidation and degradation,^59^ PbS/CdS QDs with core–shell structures were produced through a more stable CdS protective shell. A PbS/CdS core–shell structure was synthesized by cation exchange by using a microfluidic system. Figure 3a shows the XRD comparison between PbS and PbS/CdS QDs synthesized with a microfluidic system over time. Starting from 5 to 20 min, the shifted peaks from PbS main peaks (26°, 30.1°, and 43.1°) to CdS main peaks (26.7°, 30.8°, and 43.8°) are noticeable. The XRD peak widens, and the angle peaks are shifted as a result of PbS/CdS’s slightly diminished *d* spacing.^32^ Additionally, the broader peaks indicate that PbS/CdS QD peaks are composed of PbS and CdS. Because it serves as a cover shell for PbS, the observed shifted peaks to the CdS character suggest that the CdS character is more intense over the reaction. The outcomes validate the validity of the synthesis using a microfluidic system by demonstrating that the CdS QDs were able to coat the PbS QDs successfully. The core–shell formation mechanism is thought to be based on cation exchange. The substitution of Cd^2+^ for Pb^2+^ is thermodynamically advantageous because of the similar ionic radii and the difference in solubility product (*K*_sp_) values, which enable a controlled transformation while maintaining the original core structure.

**Figure 3:**
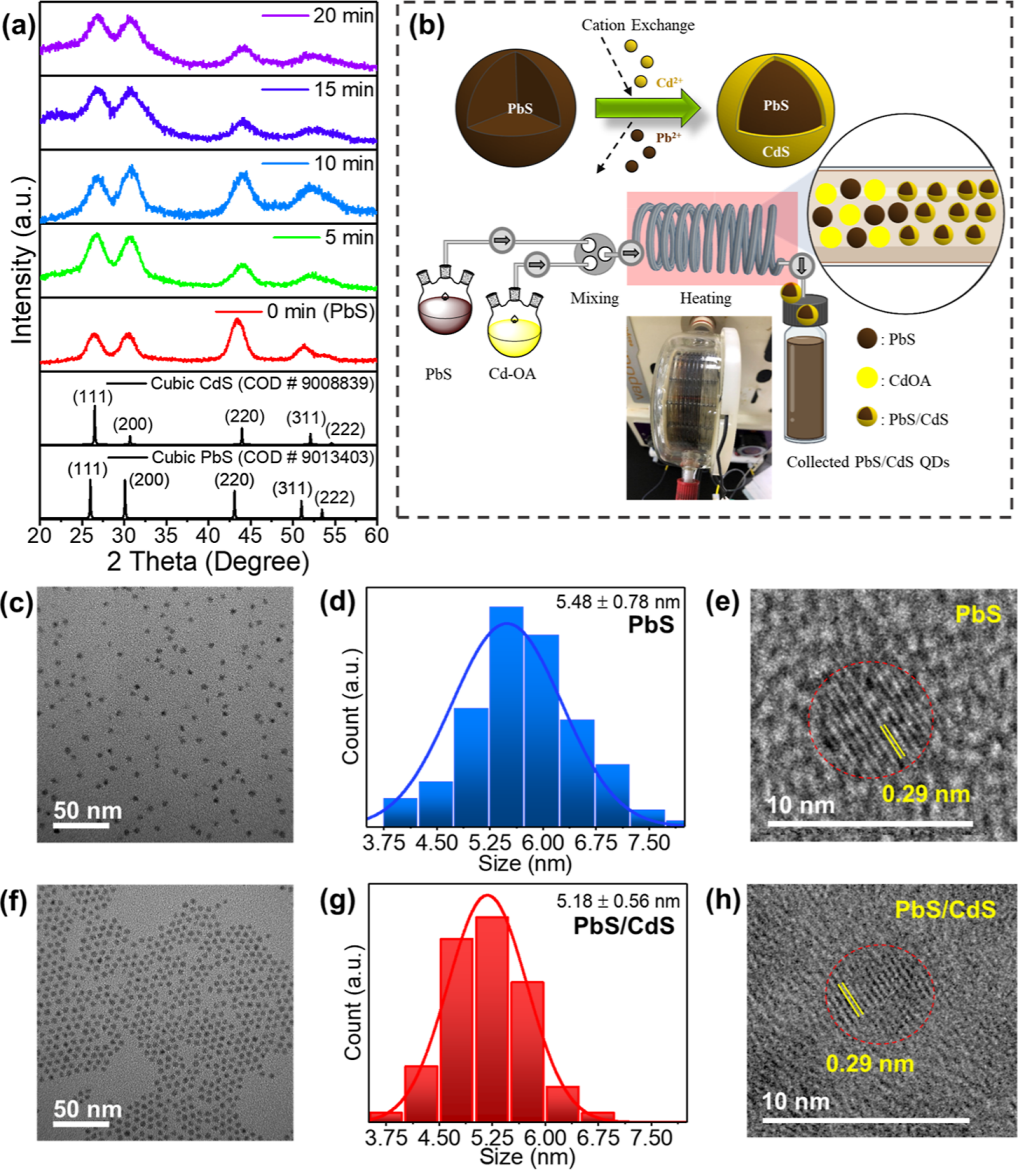
(a) XRD of the microfluidic-synthesized PbS and PbS/CdS QDs. (b) Mechanism of cation exchange during PbS coating by CdS using the microfluidic method. (c) TEM image of PbS QDs. (d) Particle size distribution diagram of PbS QDs. (e) HRTEM image of PbS QDs; a set of lattice fringes with a *d*-spacing of 0.29 nm are of the PbS (2 0 0) lattice plane. (f) TEM image of the microfluidic-synthesized PbS/CdS QDs. (g) Particle size distribution graphic of the microfluidic-synthesized PbS/CdS QDs. (h) HRTEM crystal lattice fringe image of the microfluidic-synthesized PbS/CdS QDs.

Figure 3b shows an example of cation exchange-based PbS/CdS core–shell formation. At the surface of PbS nanocrystals, Cd^2+^ cations partially replace Pb^2+^ while maintaining the S^2–^ anion structure during the cation exchange process. A PbS/CdS core–shell may eventually form as a result of this substitution, which may marginally alter the core composition or shrink it if substantial exchange takes place.^32,33^

During cation exchange, the displaced Pb^2+^ ions diffuse into the surrounding solution as solvated species and are continuously removed by microfluidic flow. Because no suitable anion is present to precipitate Pb^2+^, these ions remain dissolved rather than forming secondary phases with Cd^2+^. This continuous removal of byproducts is an inherent advantage of the microfluidic method as it minimizes unwanted side reactions and maintains cleaner reaction conditions.

TEM observation was used to analyze the QD size change and verify the PbS/CdS core–shell QD structure (Figure 3c–h). Figure 3c–e show the TEM analysis of PbS QDs before cation exchange, serving as comparison data for Figure 3f–h, which present the TEM analysis after cation exchange (PbS/CdS core–shell QDs). However, due to the nanosized thin shell layer and crystal lattices matching so well, the interface between PbS and CdS was tricky to observe intentionally. The average size of PbS QDs was observed as 5.48 ± 0.78 nm (Figure 3c,d) with a lattice fringe of 0.29 nm (Figure 3e). Compared with the TEM image of PbS/CdS after the reaction (Figure 3f,g), the average size is 5.18 ± 0.56 nm with a similar lattice fringe of 0.29 nm (Figure 3h); thus, the core–shell structure is smaller compared with that of PbS. The difference between the Pb ion radius of 0.119 nm and the Cd ion radius of 0.095 nm is about 0.24 nm, which is similar to the TEM estimate of the CdS shell thickness of 0.3 nm.^34^

The development of a CdS shell on PbS nanocrystals is frequently followed by a decrease in the particle size. Smaller crystallite domains could result from structural reorganization or fragmentation brought on by the cation exchange process, which switches out some of the Pb^2+^ with Cd^2+^. Lattice strain resulting from the mismatch between the ionic radii and lattice parameters of PbS and CdS may also cause relaxation by forming smaller, more stable crystalline regions. Sometimes the particle dimensions can be physically reduced by mild etching that occurs during the cation exchange process.

Furthermore, using eq 1, the XRD peak fwhm data from Figure 3a show that the particles’ size decreases from PbS to PbS/CdS over the microfluidic reaction time (Table S3). The calculation from the Sherrer formula tends to give a smaller size than TEM results because of the wider peaks caused by mixed PbS/CdS, which in turn causes larger fwhm. Nevertheless, the TEM and XRD data showed a progressive decrease in particle size, which supports the core–shell formation through cation exchange.

As observed by the HRTEM image in Figure 3e, a set of lattice fringes with a *d*-spacing of 0.29 nm are of the (200) lattice plane of PbS, which is consistent with the HRTEM of PbS/CdS with a *d*-spacing of 0.29 nm (Figure 3h).^38^ This result indicates that during cation exchange, the partial transformation to CdS happens on the surface of PbS and still leaves the PbS core with a smaller size. Elemental distribution of PbS/CdS analysis by EDS instrumentation shows the existence of Pb, Cd, and S in the QDs (Figure S6). The higher atomic percentage of Cd (16.74%) compared to Pb (32.76%) implies the coated structure of Pb by the Cd shell; thus, the instrument detected a higher amount of Cd in this material.

Additionally, selected area electron diffraction (SAED) was used to further analyze the samples’ structural characteristics. A polycrystalline nature with relatively low crystallinity or the presence of ultrasmall nanocrystals is indicated by the broad and diffuse diffraction rings in the SAED pattern of the pristine PbS sample (before reaction), as seen in Figure S7. On the other hand, the PbS/CdS sample’s SAED pattern (following reaction) displays more pronounced and sharp diffraction rings. This improvement in diffraction intensity and definition points to better crystallinity, which is explained by a cation exchange process that forms a crystalline CdS shell around the PbS core. The formation of a core–shell structure is supported by the appearance of more or more intense rings, which is consistent with the introduction of a new phase, most likely CdS. These findings suggest that the observed increase in crystalline order may be due to the CdS shell growing epitaxially or semiepitaxially on the PbS surface.

The PL spectrum of PbS/CdS QDs synthesized by cation exchange through the microfluidic system is shown in Figure 4a. The fluorescence and absorption spectra of CdS-coated PbS synthesized with the cation exchange method will blue-shift the peak due to excess Cd ions, resulting in cation exchange. The reduction in particle sizes of this core, as we discussed previously, results in a blue shift in the spectrum. The relationship between the reaction time and the fluorescence spectrum is shown in Figure 4b. In the initial 5 min, the fluorescence spectra have a significant blue shift, which is due to the occurrence of the cation exchange method. After the initial 5 min, extending the reaction time to 20 min resulted in a blue shift of up to 145 nm, although the change was not significant. We speculate that this is because the microfluidic system provides a fast diffusion characteristic which can quickly conduct cation exchange and let the PbS particle achieve self-limitation or self-passivation that prevent the further QDs growth in a short time.^32,39^ Therefore, there is no significant blue-shift change over time. Compared with the original PbS with a longer wavelength (1420 nm; Figure S8), the blue shift of the fluorescence spectrum with a reaction time of 5 min is about 152 nm. The microfluidic system can precisely control the reaction temperature within ±1 °C, which leads to the ability to give a blue-shift effect by regulating the reaction time in the range of approximately 150 nm at 5 min. This result signifies that the wavelength range of the blue shift is similar.

**Figure 4:**
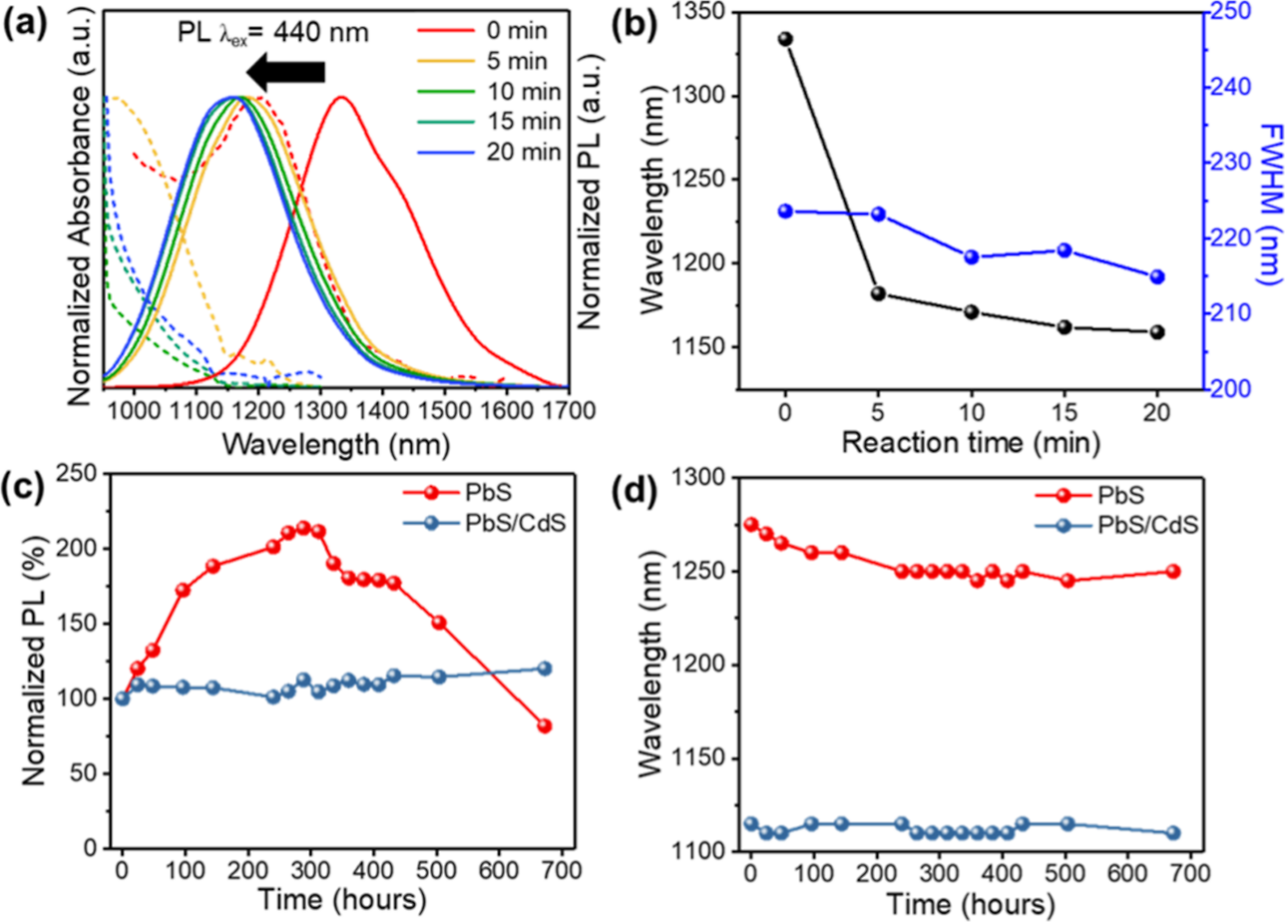
(a) PL emission and absorption spectra of PbS/CdS synthesized by the microfluidic system at different reaction times and (b) comparison of wavelength and half-width. (c) Stability of the PL spectra intensity of PbS and PbS/CdS QDs under normal conditions and their (d) peak’s wavelength change.

The bandgap of the synthesized QDs was estimated using the PL emission spectra using the relation. The emission peak positions were used to approximate the optical bandgap energies, which ranged from 0.84 to 1.04 eV for PbS QDs and from 0.93 to 1.07 eV for PbS/CdS QDs (Tables S4 and S5). These values are consistent with the expected quantum confinement effects and the slight bandgap increase upon CdS shell passivation.

Additionally, the half-width of the spectra (Figure 4b) indicates no widening with an increase of the reaction time. This result signifies that the core size distribution of the PbS/CdS core–shell structure is uniform.^36^ The time-resolved PL peak alteration was tracked over 20 min to track the reaction from PbS to PbS/CdS. The PL emission wavelength showed a noticeable blue shift from 1334 nm at 0 min to 1159 nm at 20 min, as seen in Figure S9. Using a single-exponential decay function like eq 2, this spectral evolution was well-fitted and exhibited typical first-order kinetic behavior:

2 $$y\left(t\right)=y_{0}+A_{1}.e^{-t/t1}$$

where *y*_0_ is the final stabilized emission wavelength, *A*_1_ represents the amplitude of the PL shift, and *t*1 is the time constant of the reaction. The spectral change’s exponential kinetic pattern, which is typical of ion exchange reactions, is supported by this fitting data. With a half-life of 1.64 min, the fitting produced values of *y*_0_ = 1162.63 nm, *A*_1_ = 171.29 nm, and *t*1 = 2.37 min, suggesting a quick transition into the PbS/CdS core–shell structure under the specified circumstances. The narrow size distribution and adjustable emission properties of PbS/CdS core/shell QDs are made possible by these quick and manageable kinetics.

Furthermore, PbS and PbS/CdS QDs samples synthesized by the microfluidic system were subjected to normal environmental conditions for hours to measure their stability. The PbS QDs exhibit a notable rise in PL intensity over a brief storage time, as seen in Figure 4c. This phenomenon occurs because the PbS QD surface is oxidized by the air, reducing the core size. A thin oxide layer shell is formed (PbO) on the surface, causing the passivation of the surface.^59,60^ After that, the fluorescence intensity decreases gradually, due to the presence of harmful oxides such as PbSO_3_ and PbSO_4_ in the ambient air that will also produce trap states.^61^ However, the PL intensity of PbS/CdS QDs in storage under ambient conditions has not decreased significantly, suggesting that the CdS shell can effectively protect the PbS core and has good environmental stability. From Figure 4d, the change in the PL spectra peaks of PbS QDs with time can be observed. The oxidation that results from shrinking the core size is consistent with an irreversible blue shift to lower wavelengths. The PL peak wavelength of PbS/CdS QDs was observed to have no significant blue shift by storage time, indicating that the core–shell structure can effectively protect the core.^60^ The PbS/CdS core–shell QDs exhibit a Type I band alignment, where both the conduction and valence band edges of CdS lie outside those of the PbS core. This configuration facilitates the confinement of both electrons and holes within the PbS core, promoting efficient radiative recombination and enhanced photoluminescence stability.^62^ Therefore, the microfluidic system can successfully synthesize stable PbS/CdS core–shell QDs with only a short reaction time.

Table 1 summarizes a comparison with earlier research on the synthesis of PbS/CdS core–shell QDs in order to verify our findings. The core/shell heterostructure was successfully formed, as evidenced by the photoluminescence (PL) peaks of the QDs synthesized in this work (1150–1200 nm), which fall within the usual emission range reported for PbS/CdS systems. The fwhm values, which range from 215 to 222 nm, also show a comparable size distribution and heterogeneity and are in line with values reported in the literature for materials of a similar nature.

**Table 1:** Comparison Table of the Synthesis Methods of PbS/CdS QDs and Their PL Properties

synthesis method	PL peak (nm)	fwhm (nm)	synthesis time (min)	notes	refs
microfluidic cation exchange	1150–1200	215–222	5–20	rapid reaction time	this work
continuous flow chemistry	940–1130		10–20	high PLQY 91%	^41^
room-temperature colloidal cation exchange	796–1020	152	>30	low temperature	^63^
hot injection cation exchange	1000–1500	not specified	not specified		^64^
	975–905	not specified	>40		^66^
	480–1875	46–353	minimum 120	efficient photon upconversion	^67^
	967	not specified	not specified		^68^
new photochemical approach	not specified	not specified	>15		^65^
microwave-assisted cation exchange	1300–1600	around 130–185	>20	PLQY 57%, monodisperse	^69^

In contrast to other well-established techniques, our synthesis exhibits a noticeably shorter reaction time (5–20 min). In opposition to hot-injection-based techniques like those by Liu and Huang, which frequently take longer than 40 min and lack precise control over shell growth time,^65,66^ the commonly used room-temperature colloidal cation exchange described by Durmusoglu et al. (2017) takes more than 30 min for shell formation.^63^ Similarly, Ren et al. (2013) used microwave-assisted methods that produce higher PLQYs (57%) but still have relatively broad fwhm (∼130–185 nm) and longer wavelengths (1300–1600 nm) after more than 20 min of processing.^69^ Our technique, which is based on a microfluidic cation exchange approach, has the advantages of being easy to employ, quick to process, and repeatable. In line with continuous flow chemistry techniques, our setup facilitates efficient cation exchange at a wider scale, enabling precise control over reaction parameters and faster optimization.^41^

### Core/Shell PbS/CdS QD NIR Ink Optimization for Anti-Counterfeiting Applications

When discussing inks used for inkjet printing, printability and dispersibility ought to come first. Therefore, NIR PbS/CdS QDs were dispersed in an *n*-octane solvent to obtain good dispersibility of an NIR ink. However, because viscosity effectively impacts the print performance, ink cannot be ejected smoothly during inkjet printing. The ink’s viscosity has a direct impact on the ease with which it flows past the nozzle and into the cartridge. Particularly, if the ink’s viscosity is high, insufficient ink flow results in nozzle clogging. Low ink viscosity might cause automatic liquid leakage.^50^ Since the viscosity of *n*-octane is about 0.57 mPa·s, the solvent is too dilute to eject smoothly during the inkjet process of the inkjet printer.^70^ Therefore, in this study, we utilized 1-octadecene (ODE) with a viscosity of 4.32 mPa·s. We formulated it as a binary solvent ink so that the ink can be ejected smoothly by adjusting the volume ratio of *n*-octane and ODE.

We used each 10% as a unit, and the binary solvent was used for the physical properties such as density (Tables S6 and S7), viscosity (Tables S8 and S9), and surface tension (Table S10). Overall data are delivered in Figure 5a. With the addition of ODE, the density, viscosity, and surface tension of the ink are increased. The viscosity is significantly improved from 0.57 to 3.13 mPa·s mainly because of the addition of ODE. Figure S10 displays the results of testing a binary solvent with varying volume ratios on an inkjet printer. With the addition of ODE to 40%, the ink can be issued from the inkjet printer, but there would be a case of ink breakage because the viscosity is too low. Because of the nozzle’s excessively high inkjet speed, ink builds up on it, and the print head becomes blocked.^71^ This phenomenon occurs because the NIR QD ink dots are reflected at the bottom of the print head. The incidence of inkjet ink breakage was decreased by adding 50% ODE. Compared with the addition of 40 % of ODE, the viscosity increases from 1.13 to 1.41 mPa·s. The increase in the viscosity reduces the occurrence of ink breakage. With the addition of an ODE up to 60% with a viscosity of 1.72 mPa·s, there is no ink breakage. When 70–90% ODE was added, no ink breakage occurred anymore. Based on our inkjet image experiment, the best printability ink was performed by adding 60–90% ODE.

**Figure 5:**
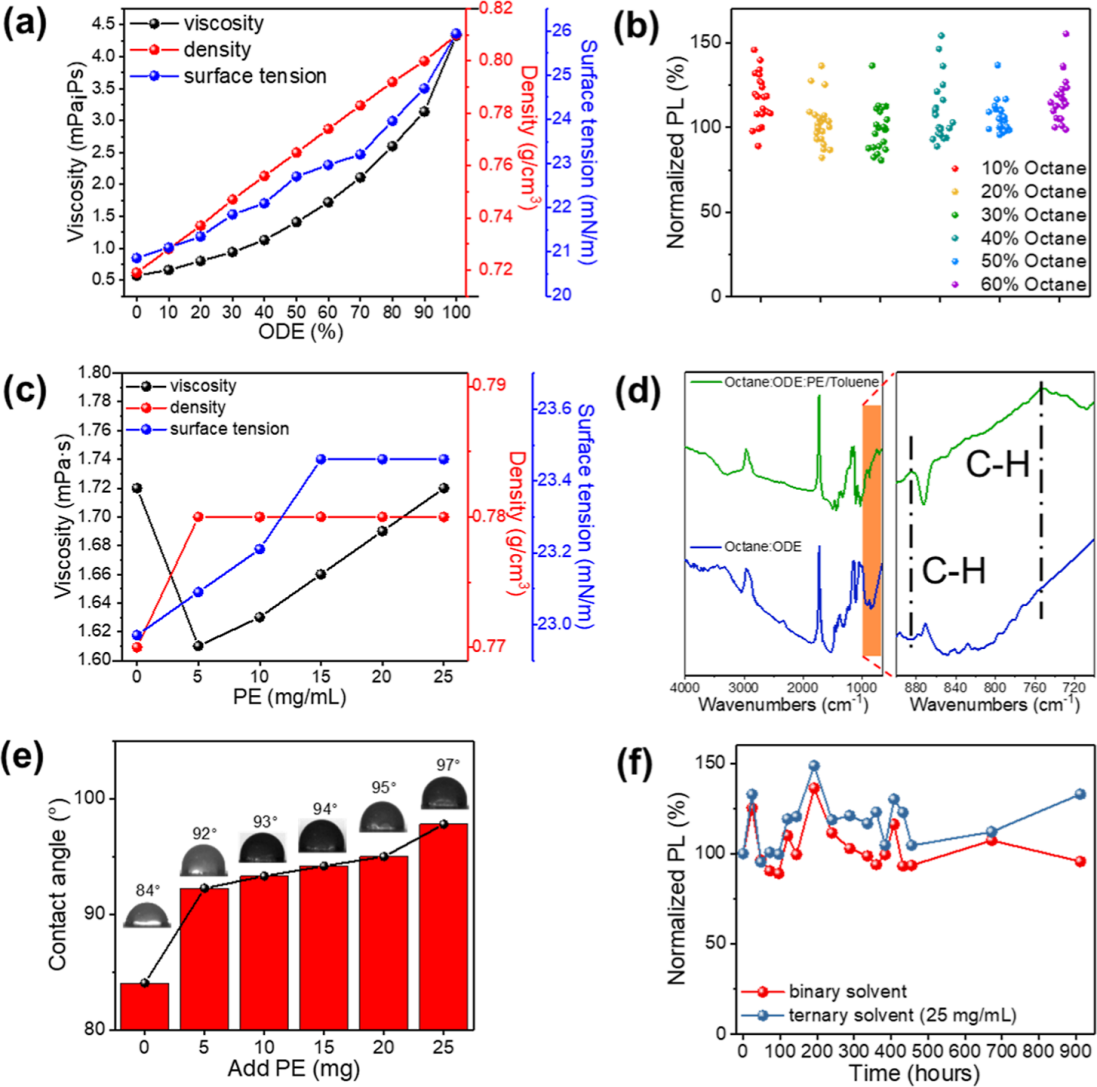
(a) The physical properties of the binary solvent, such as viscosity, density, and surface tension. (b) The fluorescence stability of the solvent with different ODE ratios. (c) The physical properties of the ternary solvent, such as viscosity, density, and surface tension. (d) FTIR spectra of binary solvents and ternary solvents. (e) Contact angle values of ternary solvents with different amounts of PE added. (f) PL stability comparison of binary and ternary solvents inks.

A general ambient PL stability test was performed on binary solvent NIR inks with different volume ratios. Figure 5b describes that NIR QDs are dissolved in binary solvents with sufficient stability in various volumes, and there is no significant decrease in strength after one month of storage. We still found a fluctuating pattern in the PL intensity that might be caused by several common factors, such as agglomeration in the new solvent or degradation by environmental factors (humidity and UV light) that may influence the QD intensity change.^72,73^ However, we believe that the change interval was normal and was not significant. The OA ligands of their NIR QDs have good dispersibility in nonpolar solvents, such as *n*-octane and ODE binary solvents. We found no significant precipitation, causing no clogging of the nozzle of the inkjet printer.

Additionally, we put the ink in the inkjet printer for inkjet printing to double A paper and tested the number of inkjets from 1 to 5 times. The PL intensity of the printed ink is shown in Figure S11a. As the number of inkjets increases, the fluorescence intensity also increases because there are more NIR QDs per unit area (Figure S11b). However, as Figure S12 illustrates, smearing will increase with the number of inkjets used. The substrate is paper, which is easy to cause the inkjet to spread outward, resulting in smearing.^74^ A colored layer can be observed, and diffusion with a transparent layer occurs. With the increase in inkjet numbers, the blooming distance in this situation is more notable. However, we found no smearing phenomenon after one inkjet; therefore, this ink does not spread on the double A paper once. The smudge phenomenon makes it easy to make the image unclear; therefore, it is the best option to select the inkjet once. Furthermore, the ODE has a boiling point of roughly 314 °C and inadequate volatility and vapor pressure. Therefore, the minimum amount of the ODE and the proportion of graphics were selected before printing. Based on this experiment, we found that the best condition for the ink was octane/ODE = 4:6, which can print smoothly and does not produce ink breakage, thus making the printed pattern clear and detectable.

The optimalization was also conducted by assessing the ink performance in inkjet printing applications for anti-counterfeiting. The prepared NIR QD ink was printed on ordinary double A paper by an inkjet printer at one time, excited by a UV-light flashlight, and photographed by an infrared camera to obtain the image. A filter was installed to block the visible light before being photographed in the NIR wavelength range. First, an image was taken with an NIR camera using a binary solvent ink with octane/ODE ratios from 1:9 to 8:2, as shown in Figure S13. The words “NTUT APCL” were used as the printed image, and ink was printed on double A paper, which is invisible to the naked eye. The area where the printed image was irradiated by a UV-light flashlight was captured by an infrared camera. Because of the low viscosity, the octane/ODE = 8:2 sample produces no image when photographed with an infrared camera. The 7:3 ratio produces some microimages, but the ink breakage was genuine as the ODE ratio increased and reduced ink breakages. At a 5:5 ratio, there is still some slight ink breakage. However, when the ratio is increased to 4:6, it prevents ink breakage. If the ODE ratio continues to improve, then there will be no ink breakage anymore.

In addition, an image was taken with an NIR camera using a binary solvent with octane/ODE ratios from 1:9 to 8:2. As shown in Figure S14, a pattern printed with the QR code is placed on the printing area of a commercially available water-based ink. Since the pattern was undetectable to the human eye, the printed image was placed in an area illuminated by a blue light flashlight and an NIR camera was used to take the picture. The situation was the same as in Figure S13. Before adding 60% ODE, the ink broke into the images as the proportion of the addition of ODE increased. The phenomenon of ink breakage will be reduced until the ratio of the ODE reaches 60%. These inkjet printing experiment results were in line with our previous statement that the best formulation of binary solvent is octane/ODE = 4:6.

After that, in order to improve the quality of the ink, we considered a paper substrate that is easily affected by water or ink, resulting in wetting and smearing. Therefore, the addition of certain polymers was used to protect the printing substrate and reduce the impact of water on inkjet images.^75^ Here, we used PE in a toluene (PE/toluene) solution as an additive to a binary solvent ink. Adding a small amount of PE/toluene to a binary solvent forms a ternary solvent. Alternative polymers such as poly­(methyl methacrylic acid) (PMMA), polystyrene, and polyvinyl pyrrolidone (PVP) were considered. However, PMMA and PVP, being more polar, can interact unfavorably with the QD surface ligands, potentially causing aggregation and emission quenching. Polystyrene, although hydrophobic, is less soluble in the selected solvent blend and can produce viscosity increases that are not favorable for inkjet printability. In contrast, PE provides good solubility in toluene, maintains a low viscosity window, and enables a controlled adjustment of surface tension.

The binary solvent octane/ODE = 4:6 is used as the standard, and the PE/toluene concentration was regulated from 5 to 25 mg/mL with each ratio of octane/ODE/PE/toluene = 4:6:0.5. The physical properties such as density (Tables S11 and S12), viscosity (Tables S13 and S14), and surface tension (Table S15) were measured by the ternary solvent and are arranged in Figure 5c. After the addition of PE/toluene, the viscosity first decreased to 1.6 mPa·s. When the amount of PE was increased to 25 mg/mL, the viscosity reached the value for the original binary solvent at 1.72 mPa·s. On the other hand, the density increased by 0.01, and there was no significant change with the increase of the addition amount. The surface tension increased with an increase in the addition amount and reached 23.46 mN/m without a noteworthy increase.

The binary and ternary solvents were inkjet-printed on paper, dried, and then analyzed by FTIR, as depicted in Figure 5d. Significant differences were observed in the peaks at 885 cm^–1^ and 754 cm^–1^ corresponding to C–H nonplanar bending vibration. This bond comes from the addition of the PE polymer, and its existence confirms the PE polymer in the ink. The binary and ternary solvents with different addition amounts of PE/toluene were inkjet-printed onto the paper (Figure 5e), and the water contact angle was measured. With the increase of the addition amount of the PE polymer from 0 to 25 mg, the water contact angle changed from 79° to 97°. The water contact angle increases significantly to 97°, indicating that the hydrophobicity of the material increases after the addition of the PE polymer. Because the C–H bond composition of PE polyethene is nonpolar, its excellent water resistance characteristics may lead to an increase in the contact angle. Therefore, this polymer addition has significantly improved the ink’s water resistance.^76^ Under the optimal condition of 25 mg/mL PE/Toluene, DI water was dropped onto the printed paper to simulate post-print water contact. Figure S15 compares the performance of Double A paper, commercial inkjet inks, binary solvents, and ternary solvents under these conditions. Double A paper only needs 10 s to let water droplets penetrate entirely. The commercial ink that was printed on double A paper takes 40 s to penetrate, but the water droplets caused significant damage to the image because the water-based ink was easily dissolved by the droplets. For the binary ink, it takes 40 min for water droplets to penetrate because the oil-based ink is nonpolar and forms a hydrophobic condition against water droplets. The ternary ink with the addition of the PE polymer increases the water droplet penetration time to 65 min, which is consistent with the measurement of the water contact angle because the addition of the PE polymer improves the hydrophobicity and further reduces the influence of water. A comparison of PL stability toward the environment of binary solvent and ternary solvent NIR QD ink is delivered in Figure 5f. Polymer addition can give better PL stability of the ink as the NIR QD material has been protected by the PE polymer on the outside, which reduces the impact of the environment.

Supporting that result, in the inkjet printing for the anti-counterfeiting experiment, the ternary solvent ink with different amounts of PE/toluene was photographed by an NIR camera, which is consistent with the measurement method of the binary solvent. As shown in Figure S16, the image can be printed smoothly after the addition of the polymer. At a ratio of 5 mg/mL, there is still slight ink breakage caused by its viscosity decrease and slightly due to toluene solvent addition. However, as the viscosity of the PE-added ink was increased, no more ink breakage was found.

Therefore, we selected octane/ODE/PE/toluene = 4:6:0.5 (PE 25 mg/mL) as an optimal NIR ink and printed with the brief description schematic shown in Figure 6a. The printed patterns on A4 paper included a barcode (4 × 2 in), a QR code (2 × 2 in), and text using Times New Roman font at size 26. The result for this optimized ink is shown in Figure 6b, where, even after being exposed to blue light, no image is displayed. It was only possible to read the “NTUT APCL” message by using an NIR camera. Additionally, as shown in Figure 6c, the NIR ink was used in place of the commercially available water-based ink to print an inkjet luminescent QR code. We further achieved NIR anti-counterfeiting identification on different substrates and a QR code that was smoothly identified by a smartphone. The NIR ink can be used for inkjet printing on various paper substrates, such as printing on commercially available double A paper (Figure 6d), commercially available black paper (Figure 6e), Kraft paper (Figure 6f), and Dowling paper (Figure 6g).

**Figure 6:**
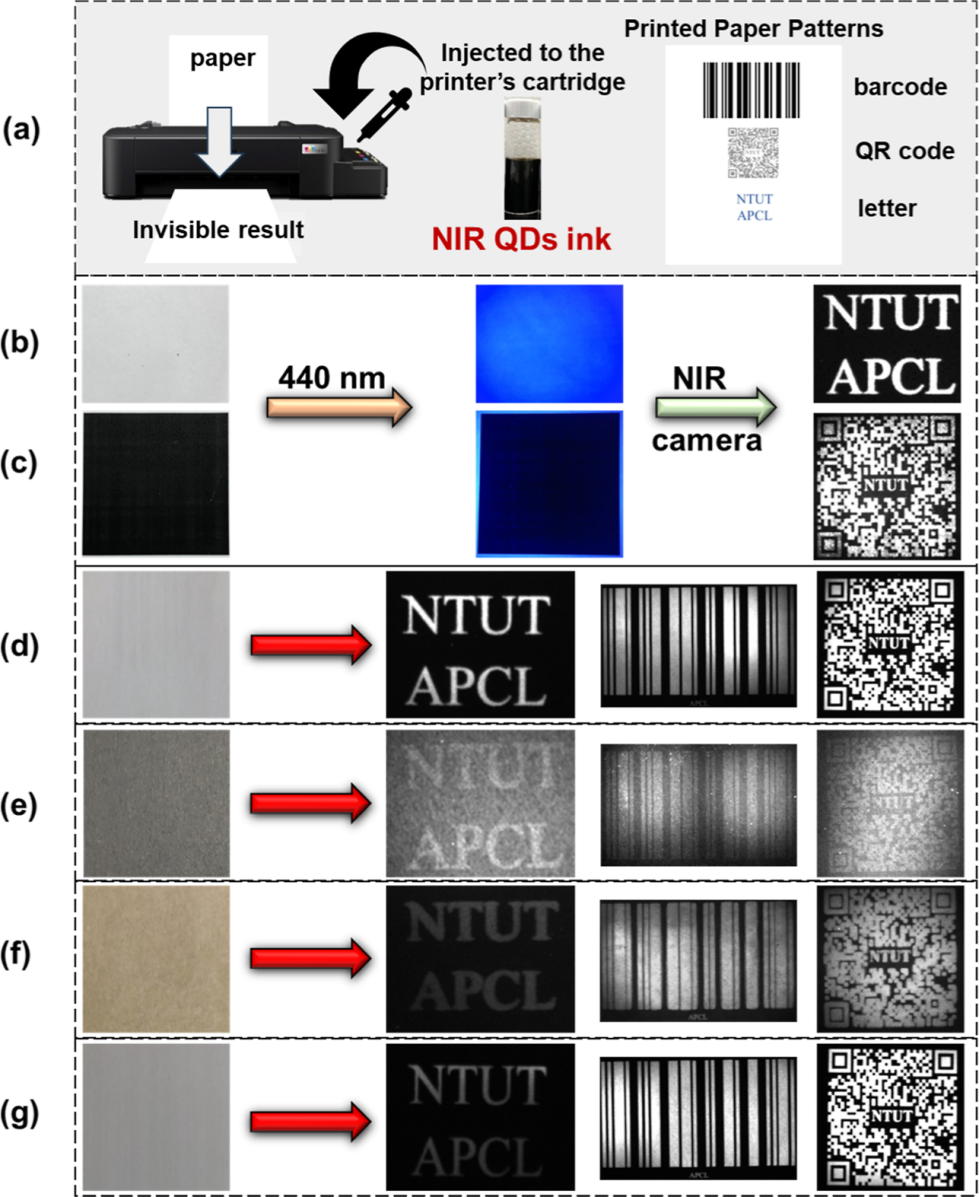
(a) Illustration of inkjet printing application of the fabricated NIR ink. (b) Photograph of double A paper printed with “NTUT APCL” in a luminous pattern under 440 nm blue light and the image taken by an infrared camera. (c) The light-emitting pattern is printed as the QR code and captured by an NIR camera under 440 nm blue light. Light-emitting pattern printed on (d) double A paper, (e) commercial black paper, (f) Kraft paper, and (g) Dowling paper captured with an NIR camera under 440 nm blue light.

The adhesive force and anti-desorption properties of QD inks are indeed critical for their practical deployment in optoelectronic and anti-counterfeiting applications. In our work, we addressed these aspects through the demonstration of QD ink compatibility across various paper substrates, including A4 paper, black paper, Kraft paper, and Dowling paper. Each of these substrates presents different surface morphologies, porosities, and chemical functionalities, all of which influence the adhesive interaction with the ink formulation. Among them, Kraft paper has more pores on the surface, resulting in imposing permeability, which is easy to cause the relative expansion of the image.^77^

The drying time of the QD ink varies slightly with substrate porosity, but because the viscosity, density, and surface tension were tuned to match those of commercial inkjet inks, the absorption and drying behavior are comparable, becoming touch-dry within seconds on standard papers. Overall, the developed ternary solvent NIR ink can be successfully inkjet-printed on commercially available inkjet printers. After inkjet-printing on different paper substrates, we also achieved the NIR emission effect in anti-counterfeiting identification to achieve a more concealed effect.

## Conclusions

According to this study, a microfluidic system can synthesize PbS and PbS/CdS to produce large-scale, continuous NIR QDs with fixed quality and tunable spectral properties. Among them, the PL emission wavelength of PbS can be from around 1200 to 1600 nm and is lower than that of the usual batch synthesis method. It only takes 5 min to synthesize the PbS/CdS core–shell structure, which is more stable than PbS and enhances the blue-shift phenomenon. The developed ternary solvent (octane/ODE/PE/toluene) NIR ink has excellent stability and a nonpolar hydrophobic effect. The contact angle reaches 97° and can last for 65 min under water droplets. The ink can be successfully printed on commercially available inkjet printers and does not produce ink breakage. The characteristics of NIR emission, which cannot be seen with the naked eye, may provide a significant advantage in highly concealed anti-counterfeiting development.

## Supplementary Material



## Notes

Detailed information about the calculations, XRD analyses, PL optical spectra, HRTEM images, and inkjet printing application with its anti-counterfeiting experiments (PDF)
