# Supplementary material for: Emerging Inkjet-Compatible Anti-Counterfeiting Inks Based on Microfluidic-Synthesized NIR PbS/CdS Quantum Dots
Source: JACS Au. 2025 Sep 18;5(10):4856–69. doi: 10.1021/jacsau.5c00787 (PMC12569701; doi:10.1021/jacsau.5c00787)
Supplement: Supplementary file 1 [file au5c00787_si_001.pdf]

Supporting Information

Emerging Inkjet-Compatible Anti-Counterfeiting Inks Based on Microfluidic-Synthesized NIR PbS/CdS Quantum Dots

Andi Magattang Gafur Muchlis [a,†], Chong-Ci Hu [a,†], Hoang-Duy Nguyen [c,†], Ramadhass Keerthika Devi [a], Yi-Ting Tsai [a], Yu Chun Lee [d], Chun Che Lin [a,b,*]

[a] Institute of Organic and Polymeric Materials, National Taipei University of Technology, Taipei, 10608, Taiwan.

[b] Research and Development Center for Smart Textile Technology, National Taipei University of Technology, Taipei, 10608, Taiwan.

[c] Institute of Advanced Technology, Viet Nam Academy of Science and Technology, Hochiminh City 700000, Viet Nam.

[d] Lextar Electronics Corporation, Miaoli, 350402, Taiwan.

[†] These authors contributed equally.

* Corresponding author, E-mail: cclin0530@mail.ntut.edu.tw

Table S1. Parameters and calculation result of particle size based on XRD FWHM peaks of PbS QDs synthesized using microfluidic synthesis.

Temperature (°C)	B at 26° Peak (rad)	B at 30.1° Peak (rad)	B at 43.1° Peak (rad)	Average Particle Size (D [nm])
90	0.0323	0.0315	0.0331	4.69
100	0.0305	0.0303	0.0291	5.05
110	0.0274	0.0293	0.0337	5.06
120	0.0288	0.0306	0.0313	5.01
130	0.0333	0.0317	0.0281	4.91
140	0.0267	0.0260	0.0299	5.52
150	0.0257	0.0256	0.0313	5.54

Table S2. Parameters and calculation result of particle size based on XRD FWHM peaks of PbS QDs synthesized using batch synthesis as comparison.

Temperature (°C)	B at 26° Peak (rad)	B at 30.1° Peak (rad)	B at 43.1° Peak (rad)	Average Particle Size (D [nm])
90	0.0301	0.0284	0.0306	5.21
100	0.0308	0.0294	0.0317	5.10
110	0.0269	0.0304	0.0277	5.51
120	0.0289	0.0305	0.0305	5.24
130	0.0325	0.0317	0.0268	5.13
140	0.0277	0.0290	0.0265	5.72
150	0.0241	0.0342	0.0211	6.22

Table S3. Parameters and calculation result of particle size based on XRD FWHM peaks of PbS/CdS QDs synthesized using microfluidic synthesis.

Reaction Time (minutes)	B at 26.7° Peak (rad)	B at 30.8° Peak (rad)	B at 43.8° Peak (rad)	Average Particle Size (D [nm])
0	0.0288	0.0306	0.0313	5.01
5	0.0438	0.035	0.0709	3.29
10	0.0527	0.0482	0.0747	2.67
15	0.0766	0.0555	0.0855	2.15
20	0.0852	0.051	0.0812	2.20

Table S4. Conversion of PL emission peak of PbS synthesized using microfluidic method into bandgap energy (E_g).

Reaction Temperature (°C)	PL peak (nm)	E_g (eV)
90	1195	1.04
100	1249	0.99
110	1315	0.94
120	1346	0.92
130	1442	0.86
140	1464	0.85
150	1481	0.84

Table S5. Conversion of PL emission peak of PbS/CdS synthesized using microfluidic method into bandgap energy (E_g).

Reaction Time (minutes)	PL peak (nm)	E_g (eV)
0	1334	0.93
5	1182	1.05
10	1171	1.06
15	1162	1.07
20	1152	1.07

Table S6. The weight of binary solvents that were measured in different volume ratios with a pycnometer (water as a standard at 24°C).

**Reference standard**	**Empty bottle weight (g)**	**Standard + empty bottle weight*1 (g)**	**Standard + empty bottle weight*2 (g)**	**Standard + empty bottle weight*3 (g)**	**Average (g)**	**Standard weight (g)**
Water	10.4513	20.7342	20.7313	20.7393	20.7349	10.2836
**Octane : ODE**	**Empty bottle weight (g)**	**Sample + empty bottle weight*1 (g)**	**Sample + empty bottle weight*2 (g)**	**Sample + empty bottle weight*3 (g)**	**Average (g)**	**Standard weight (g)**
Octane	10.4510	17.6373	17.6387	17.6366	17.6375	7.1865
9:1	10.4516	17.7354	17.7305	17.7312	17.7324	7.2808
8:2	10.4512	17.8268	17.8246	17.8210	17.8241	7.3729
7:3	10.4512	17.9204	17.9196	17.9203	17.9201	7.4689
6:4	10.4510	18.0094	18.0156	18.0163	18.0138	7.5628
5:5	10.4511	18.1043	18.1068	18.1049	18.1053	7.6542
4:6	10.4510	18.1940	18.1977	18.1900	18.1939	7.7429
3:7	10.4510	18.2761	18.2763	18.2764	18.2763	7.8253
2:8	10.4510	18.3663	18.3682	18.3704	18.3683	7.9073
1:9	10.4512	18.4536	18.4546	18.4540	18.4541	8.0029
ODE	10.4511	18.5503	18.5512	18.5493	18.5502	8.0991

Table S7. The density values which derived from the weights from Table S4.

**Octane : ODE**	**Density (g/cm^3)**
Octane	0.6969
9:1	0.7060
8:2	0.7150
7:3	0.7243
6:4	0.7334
5:5	0.7423
4:6	0.7509
3:7	0.7589
2:8	0.7668
1:9	0.7761
ODE	0.7854

Table S8. The elapsed time of the liquid flows of binary solvents that were measured in different volume ratios with a Ostwald viscometer (water as a standard at 24°C).

**Reference standard**	***1 (s)**	***2 (s)**	***3 (s)**	**Average (s)**
Water	121	122	122	121.7
**Octane : ODE**	***1 (s)**	***2 (s)**	***3 (s)**	**Average (s)**
Octane	100	99	100	99.7
9:1	113	113	113	113.0
8:2	136	137	136	136.3
7:3	157	157	157	157.0
6:4	188	189	187	188.0
5:5	232	231	230	231.0
4:6	277	280	279	278.7
3:7	340	337	337	338.0
2:8	411	410	411	410.7
1:9	491	488	493	490.7
ODE	667	670	669	668.7

Table S9. The viscosity values derived from the elapsed time of the sample liquid flow from Table S6.

Octane : ODE	Viscosity (mPa·s)
Octane	0.5726
9:1	0.6575
8:2	0.8031
7:3	0.9371
6:4	1.1363
5:5	1.4131
4:6	1.7247
3:7	2.1139
2:8	2.5954
1:9	3.1385
ODE	4.3283

Table S10. The surface tension values of binary solvents that were measured in different volume ratios with a surface tension meter (water as a standard at 24°C).

Octane : ODE	Surface Tension (mN/m)
Water	72
Octane	20.86
9:1	21.10
8:2	21.35
7:3	21.85
6:4	22.10
5:5	22.71
4:6	22.97
3:7	23.21
2:8	23.96
1:9	24.70
ODE	25.94

Table S11. The weight of ternary solvents that were measured in different amounts of PE with a pycnometer (water as a standard at 24°C).

**Octane : ODE : PE/Toluene**	**Empty bottle weight (g)**	**Sample + empty bottle weight*1 (g)**	**Sample + empty bottle weight*2 (g)**	**Sample + empty bottle weight*3 (g)**	**Average (g)**	**Standard weight (g)**
Octane	10.4510	17.6373	17.6387	17.6366	17.6375	7.1865
ODE	10.4511	19.3408	19.3346	19.3348	18.5502	8.0991
Toluene	10.4511	19.3408	19.3346	19.3348	19.3367	8.8856
4:6	10.4510	18.1940	18.1977	18.1900	18.1939	7.5509
4:6:0.5 (5 mg)	10.4511	18.2456	18.2405	18.2418	18.2426	7.7915
4:6:0.5 (10 mg)	10.4510	18.2446	18.2390	18.2402	18.2413	7.7903
4:6:0.5 (15 mg)	10.4512	18.2422	18.2364	18.2366	18.2384	7.7872
4:6:0.5 (20 mg)	10.4512	18.2378	18.2354	18.2342	18.2358	7.7846
4:6:0.5 (25 mg)	10.4512	18.2327	18.2331	18.2308	18.2322	7.7810

Table S12. The density values which derived from the weights from Table S7.

**Octane : ODE : PE/Toluene**	**Density (g/cm^3)**
Octane	0.6969
ODE	0.7854
Toluene	0.8617
4:6	0.7509
4:6:0.5 (5 mg)	0.7556
4:6:0.5 (10 mg)	0.7555
4:6:0.5 (15 mg)	0.7552
4:6:0.5 (20 mg)	0.7549
4:6:0.5 (25 mg)	0.7546

Table S13. The elapsed time of the liquid flows of ternary solvents that were measured in different amounts of PE with an Ostwald viscometer (water as a standard at 24°C).

Octane : ODE : PE/Toluene	*1 (s)	*2 (s)	*3 (s)	average (s)
Octane	100	99	100	99.7
ODE	667	670	669	668.7
Toluene	91	91	91	91.0
4:6	277	280	279	278.7
4:6:0.5 (5 mg)	256	258	260	258.0
4:6:0.5 (10 mg)	262	262	262	262.0
4:6:0.5 (15 mg)	264	268	266	266.0
4:6:0.5 (20 mg)	271	273	272	272.0
4:6:0.5 (25 mg)	277	278	276	277.0

Table S14. The viscosity values which are derived from the elapsed time of the sample liquid flows from Table S9.

Octane : ODE : PE/Toluene	Viscosity (mPa·s)
Octane	0.5726
ODE	4.3283
Toluene	0.6312
4:6	1.7247
4:6:0.5 (5 mg)	1.6066
4:6:0.5 (10 mg)	1.6308
4:6:0.5 (15 mg)	1.6555
4:6:0.5 (20 mg)	1.6989
4:6:0.5 (25 mg)	1.7226

Table S15. The surface tension values of ternary solvents that were measured in different volume amounts of PE with a surface tension meter (water as a standard at 24°C).

Octane : ODE : PE/Toluene	Surface tension (mN/m)
Water	72
Octane	20.86
ODE	25.94
Toluene	28.55
4:6	22.97
4:6:0.5 (5 mg)	23.21
4:6:0.5 (10 mg)	23.46
4:6:0.5 (15 mg)	23.46
4:6:0.5 (20 mg)	23.46
4:6:0.5 (25 mg)	23.46

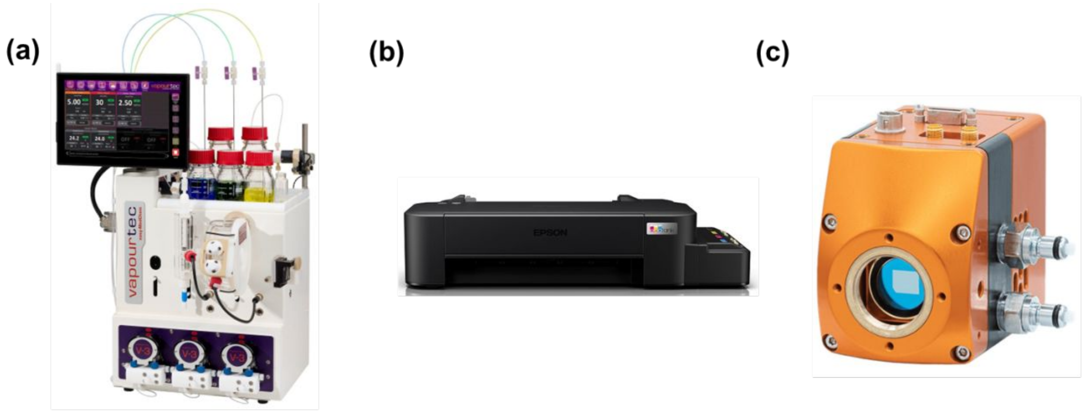

Figure S1. Photographs of instruments used in this work. (a) Vapourtec easy-MedChem microfluidic reactor system used to synthesis PbS and PbS/CdS core-shell QDs via microfluidic method, (b) EPSON L121 inkjet printer used to apply the formulated NIR QDs inks to print out designed patterns for anticounterfeiting applications, and (c) Ninox 640II VIS-SWIR camera used to captured NIR emition printed patterns.

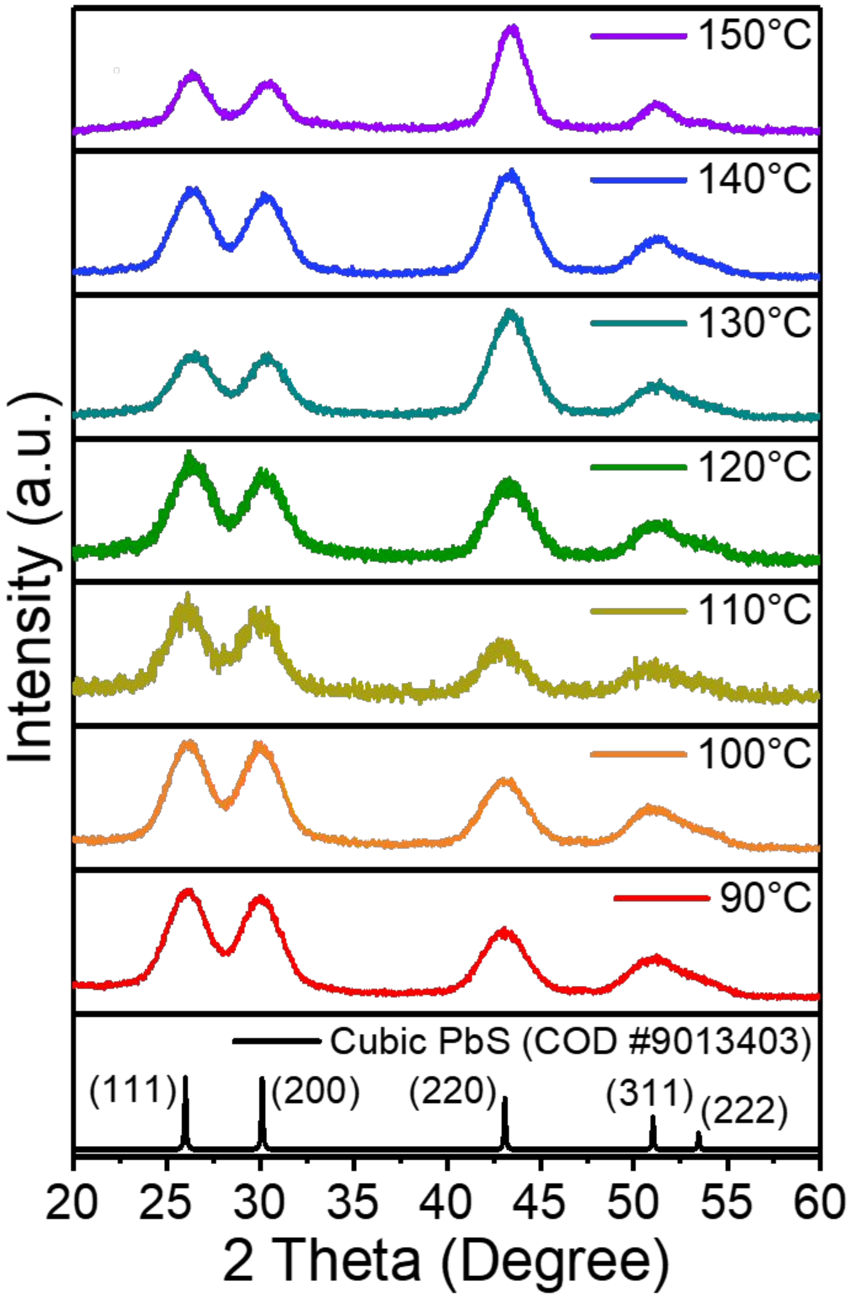

Figure S2. XRD patterns of PbS synthesized by batch hot injection method with different temperatures (90°C to 150°C).

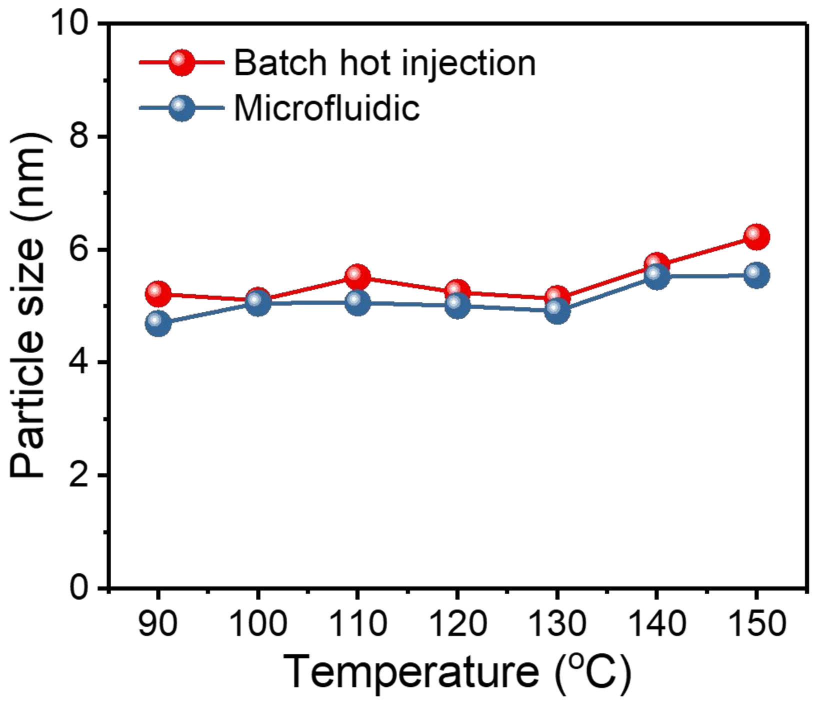

Figure S3. PbS QDs particle size comparison using batch hot injection and microfluidic method.

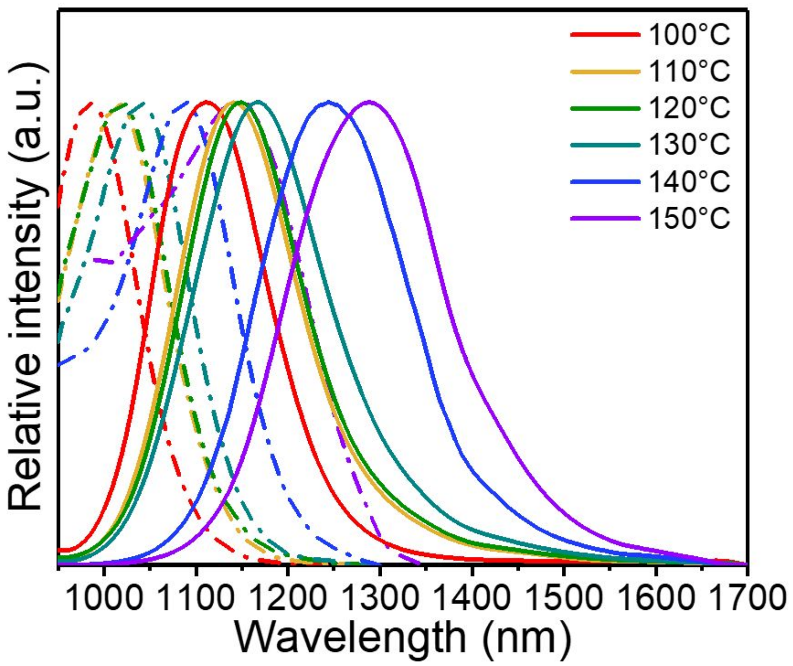

Figure S4. PL emission and absorption spectra of PbS synthesized at 100°C to 150°C synthesis temperature by batch hot injection.

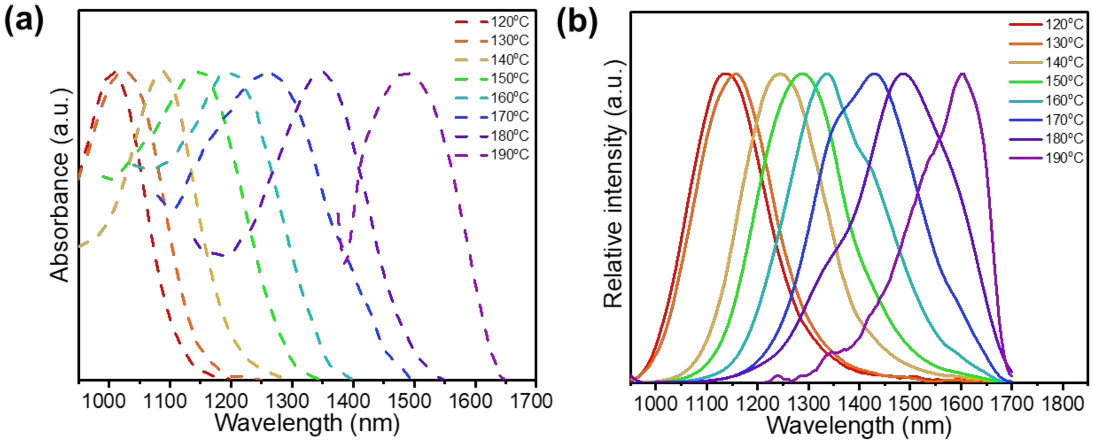

Figure S5. PL emission and absorption spectra of PbS synthesized at 120°C to 190°C synthesis temperature by batch hot injection.

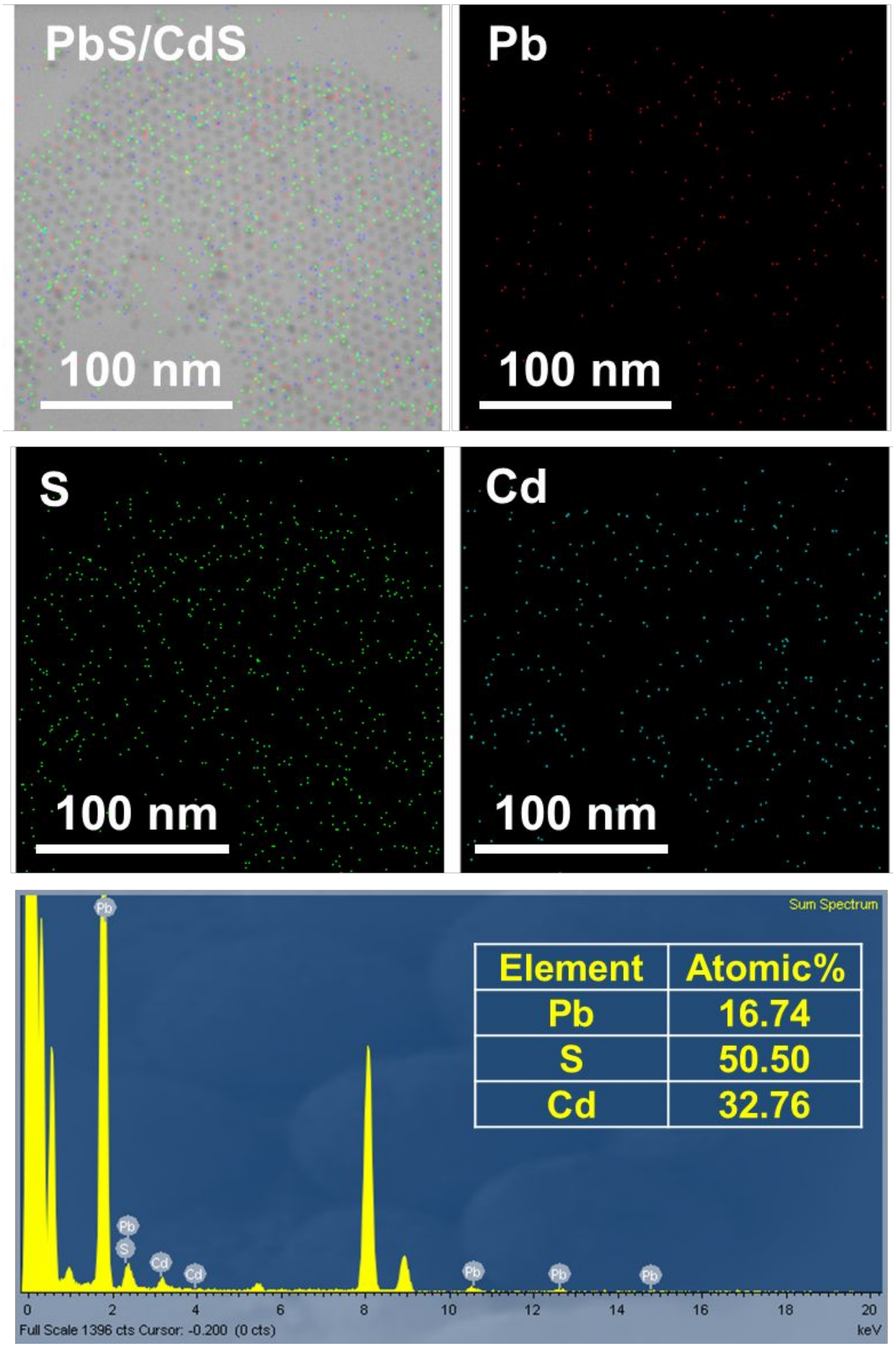

Figure S6. PbS/CdS QDs EDS images of elemental distribution.

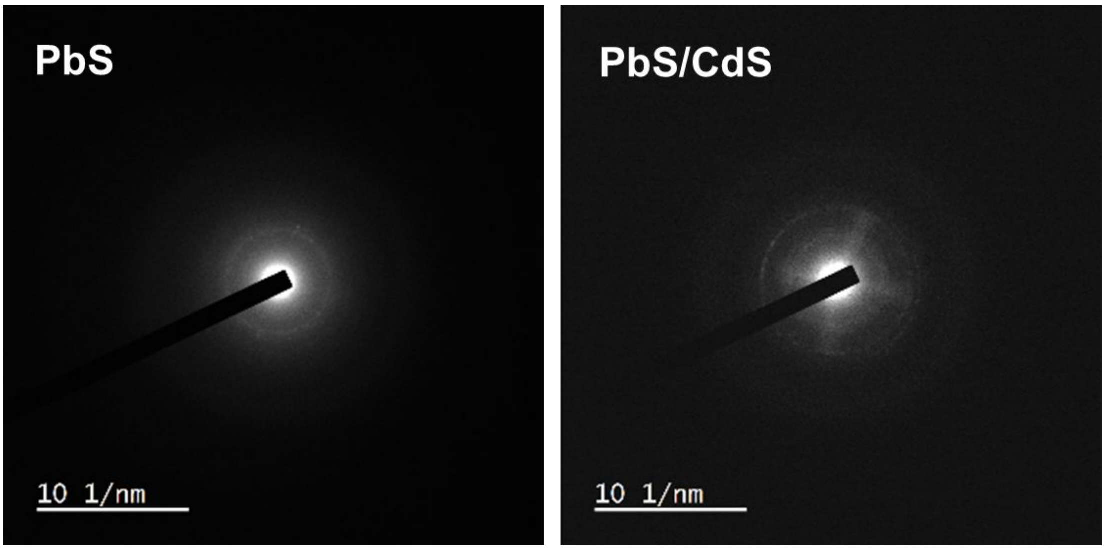

Figure S7. Selected area (electron) diffraction (SAED) pattern comparison of PbS QDs (left) and PbS/CdS QDs (right).

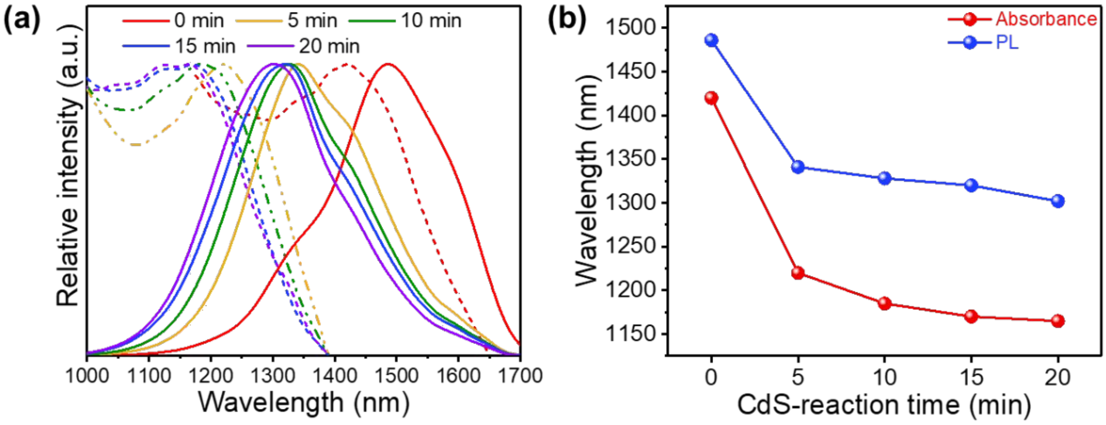

Figure S8. (a) PL absorption and emission spectra of PbS/CdS QDs synthesized by a microfluidic system from PbS QDs with an absorption wavelength of 1420 nm at different reaction times. (b) Trend plot of absorption versus emission wavelengths.

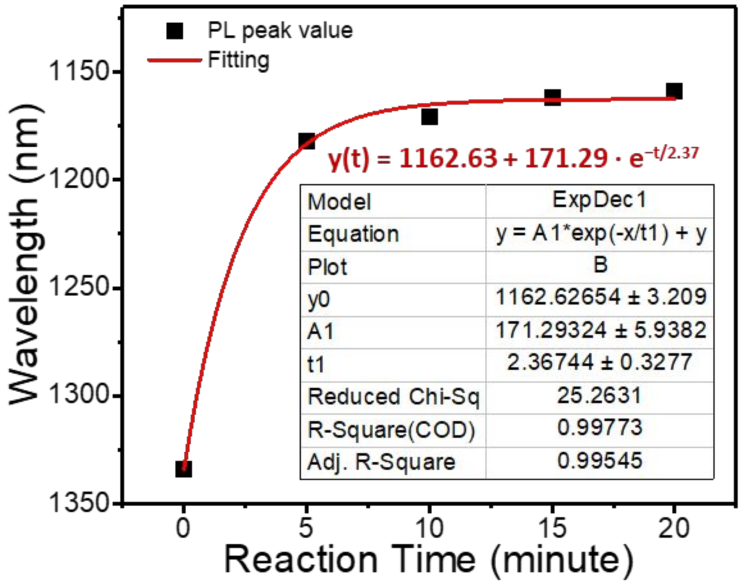

Figure S9. Fitting graphic of time-resolved PL peaks change of microfluidic synthesized PbS/CdS QDs.

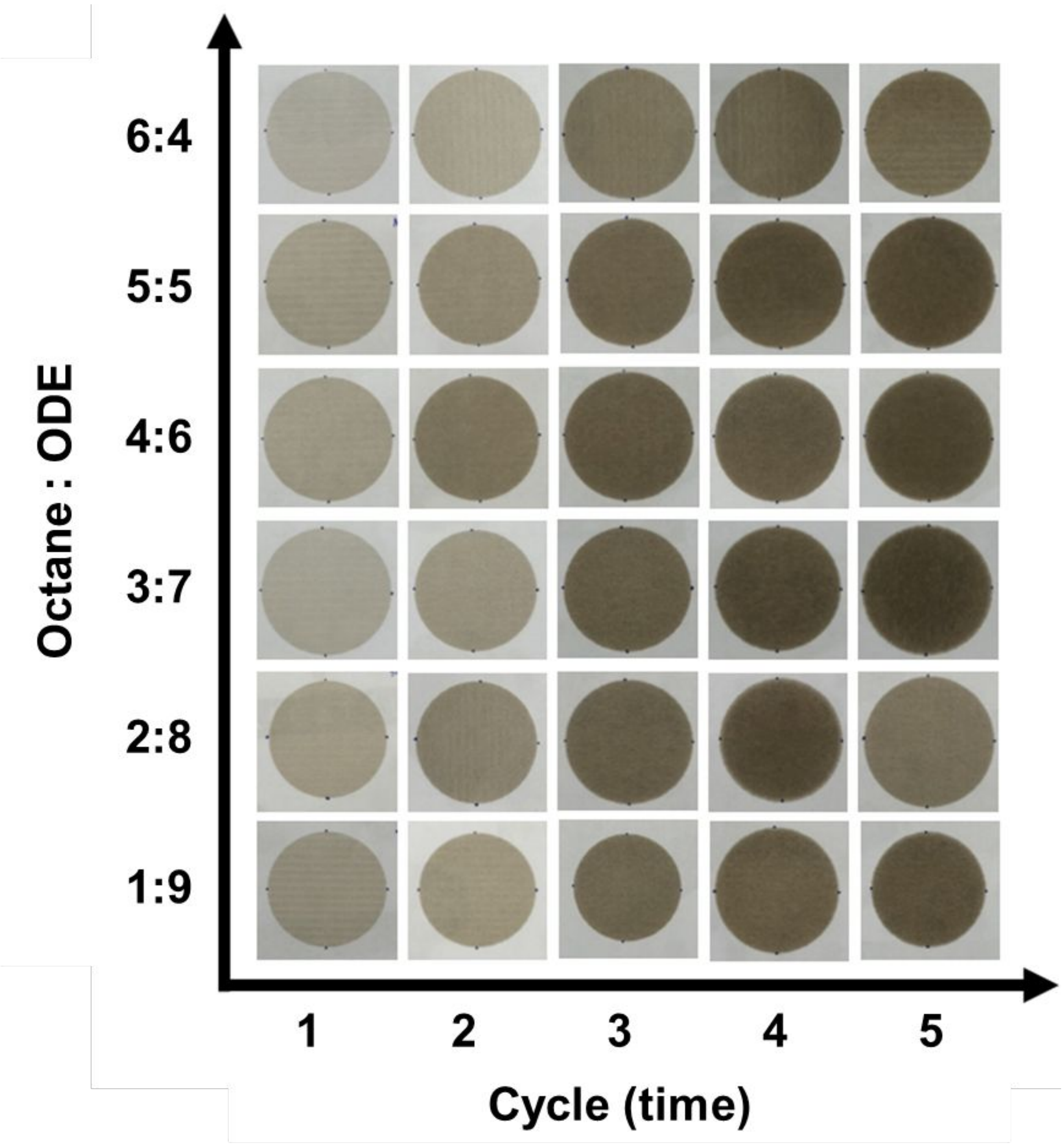

Figure S10. The actual inkjet diagram of the binary solvent ink with different volume ratios from Octane:ODE = 1:9 to 6:4 and printing times from 1 to 5 times.

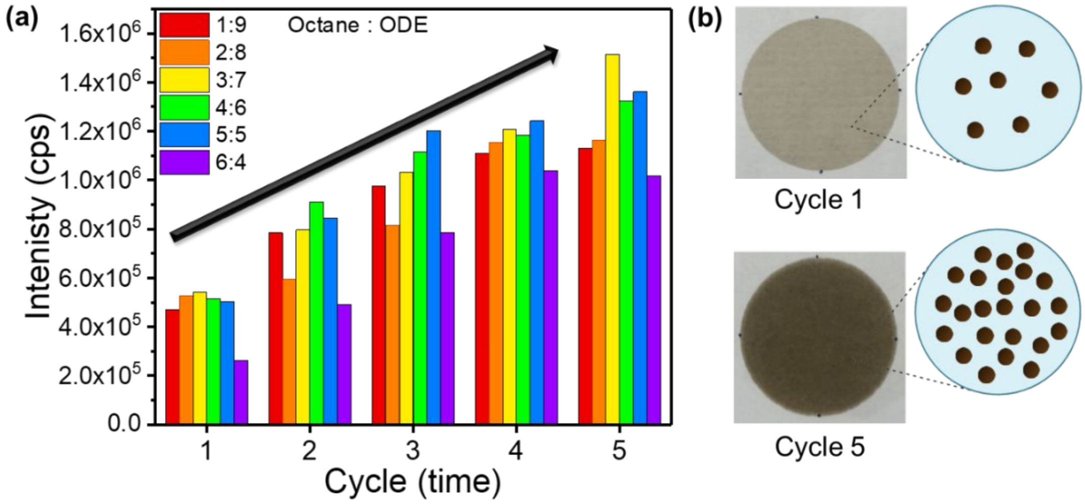

Figure S11. (a) PL emission intensities of binary solvents with different volume ratios from Octane:ODE = 1:9 to 6:4 and printing times from 1 to 5 times. (b) Illustration of different amounts of NIR QDs under different inkjet printing times at the unit area.

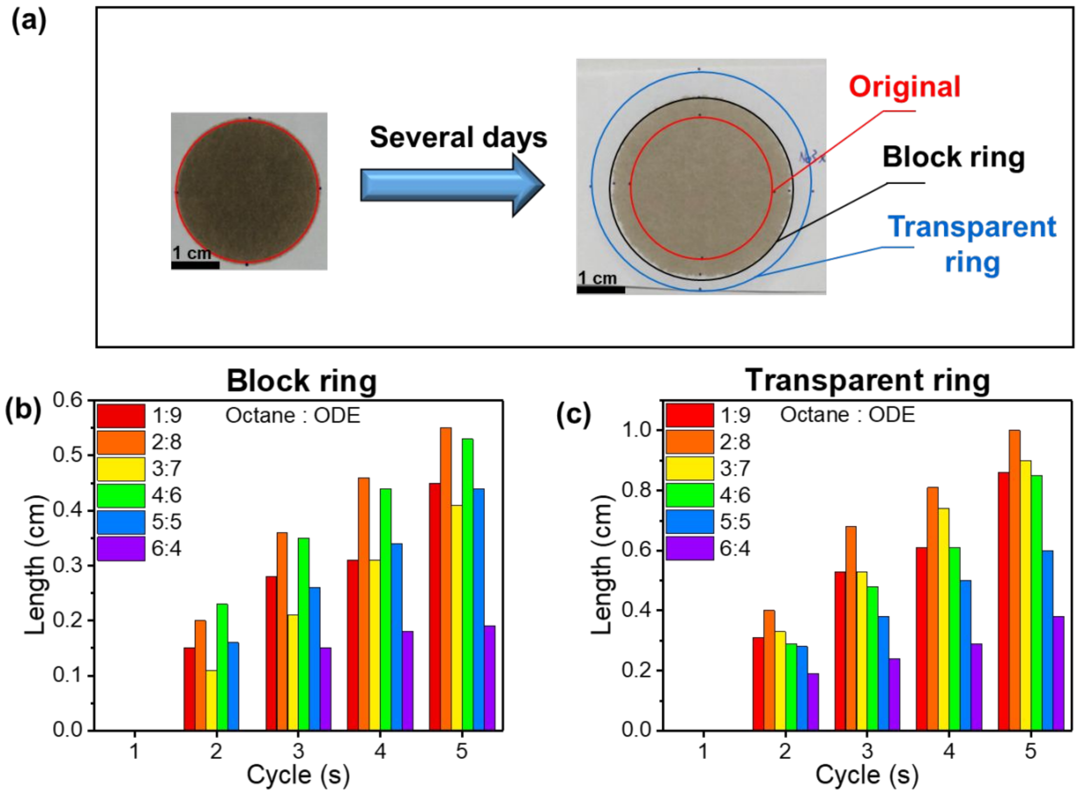

Figure S12. (a) The photograph of the NIR QDs ink that was printed too many times on the paper. After one day, there will be a phenomenon of outward diffusion and smearing, and there will be color circles and transparent circles. (b) The diffusion and blooming distance of the black circles of the binary solvents with different volume ratios in the inkjet 1 to 5 times. (c) Diffusion blooming distance of the transparent circle.

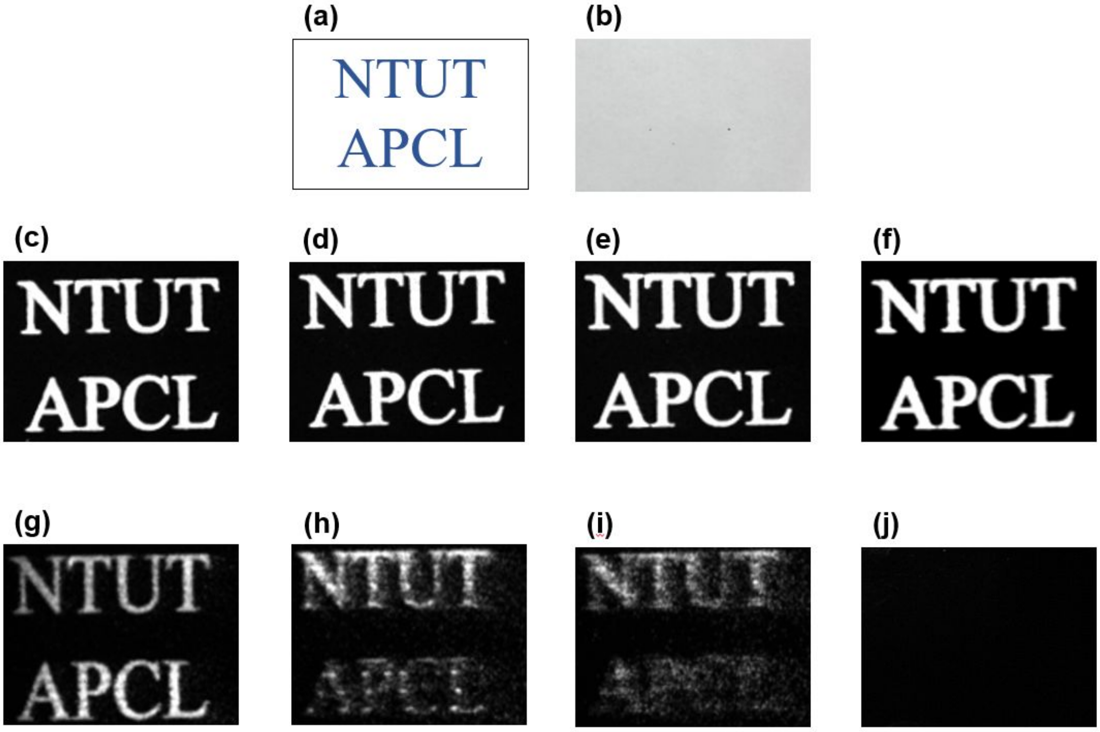

Figure S13. The photograph of NIR QDs inks with different volume ratios of Octane and ODE performance that were used for inkjet printing and the pattern was captured by an NIR camera. (a) "NTUT APCL" letters as inkjet pattern, (b) the image of inkjet on paper under visible light, different ratios of inkjet Octane:ODE (c) 1:9, (d) 2:8, (e) 3:7, (f) 4:6, (g) 5:5, (h) 6:4, (i) 7:3, and (j) 8:2 captured by an NIR camera.

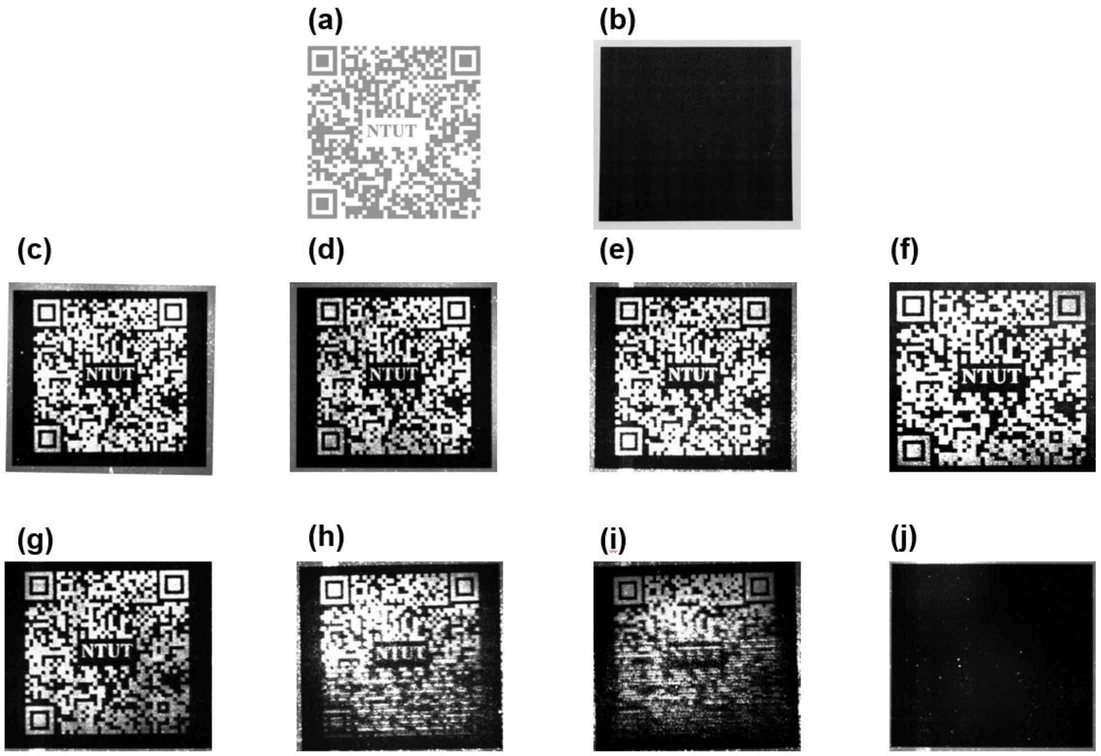

Figure S14. The photograph of NIR QDs inks with different volume ratios of Octane and ODE performance that were used for inkjet printing and the pattern was captured by an NIR camera. (a) QR code with NIR QDs ink on black printed graphic as inkjet image, (b) the image of inkjet on paper under visible light different, ratios of inkjet Octane:ODE (c) 1:9, (d) 2:8, (e) 3:7, (f) 4:6, (g) 5:5, (h) 6:4, (i) 7:3, and (j) 8:2 captured by an NIR camera.

	Double A	Commercial inks	Octane : ODE	Octane : ODE : PE/Toluene
Original	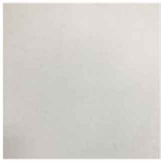	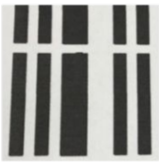	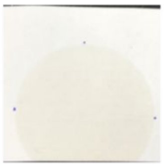	
Drop DI water	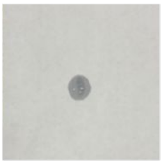	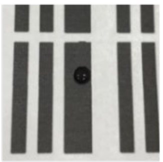	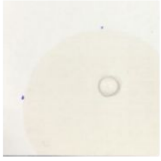	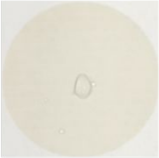
Penetration	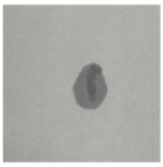	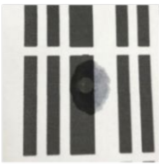	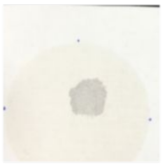	
Time (min)	0.166 min	0.666 min	40 min	65 min

Figure S15. The comparison of commercially available black ink usage, binary solvent ink, and ternary solvent ink with the influence of DI water droplet.

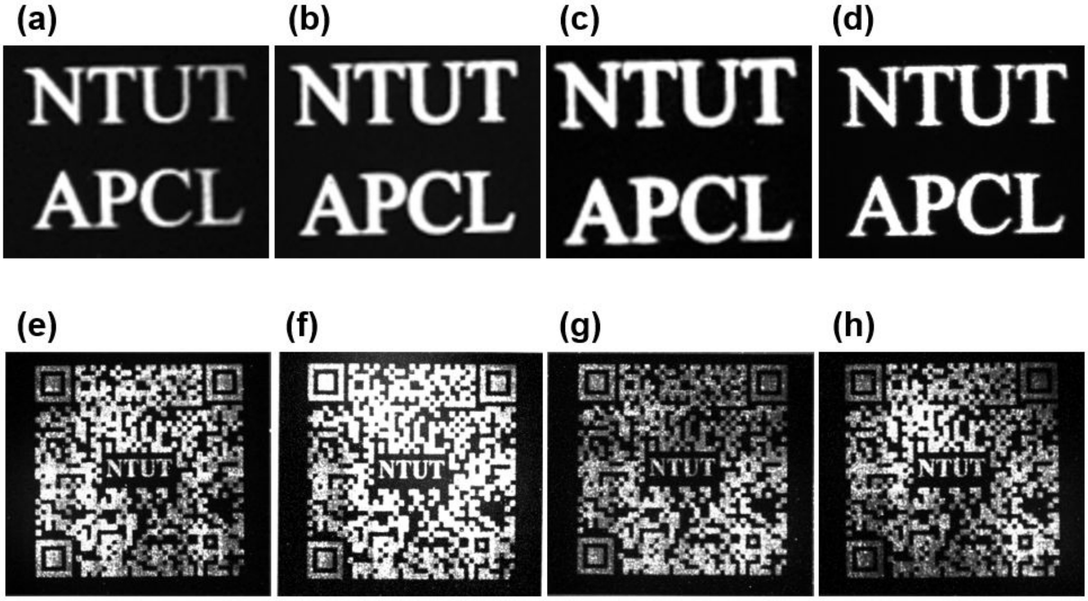

Figure S16. Inkjet graphics with "NTUT APCL" letters on the paper that the images were taken by an NIR camera using Octane:ODE:PE/Toluene = 4:6:0.5 ink with different amounts of PE added, (a) 5 mg/mL, (b) 10 mg/mL, (c) 15 mg/mL, and (d) 20 mg/mL on the paper that the images were taken by an NIR camera using Octane:ODE:PE/Toluene = 4:6:0.5 ink with different amount of PE added, (e) 5 mg/mL, (f) 10 mg/mL, (g) 15 mg/mL, and (h) 20 mg/mL.
